# Proteomic and Phosphoryproteomic Investigations Reveal that Autophagy-Related Protein 1, a Protein Kinase for Autophagy Initiation, Synchronously Deploys Phosphoregulation on the Ubiquitin-Like Conjugation System in the Mycopathogen Beauveria bassiana

**DOI:** 10.1128/msystems.01463-21

**Published:** 2022-02-08

**Authors:** Hai-Yan Lin, Jin-Li Ding, Yue-Jin Peng, Ming-Guang Feng, Sheng-Hua Ying

**Affiliations:** a Institute of Microbiology, College of Life Sciences, Zhejiang University, Hangzhou, China; University of California, Davis

**Keywords:** autophagy-related kinase, conidiogenesis, filamentous fungus, fungal development, phosphoproteomic analysis, phosphorylation activity, ubiquitin-like conjugation system

## Abstract

Autophagy is a conserved intracellular degradation mechanism in eukaryotes and is initiated by the protein kinase autophagy-related protein 1 (Atg1). However, except for the autophosphorylation activity of Atg1, the target proteins phosphorylated by Atg1 are largely unknown in filamentous fungi. In Beauveria bassiana (a filamentous insect-pathogenic fungus), Atg1 is indispensable for autophagy and is associated with fungal development. Comparative omics-based analyses revealed that B. bassiana Atg1 (BbAtg1) has key influence on the proteome and phosphoproteome during conidiogenesis. In terms of its physiological functions, the BbAtg1-mediated phosphoproteome is primarily associated with metabolism, signal transduction, cell cycle, and autophagy. At the proteomic level, BbAtg1 mainly regulates genes involved in protein synthesis, protein fate, and protein with binding function. Furthermore, integrative analyses of phosphoproteomic and proteomic data led to the identification of several potential targets regulated by BbAtg1 phosphorylation activity. Notably, we demonstrated that BbAtg1 phosphorylated BbAtg3, an essential component of the ubiquitin-like conjugation system in autophagic progress. Our findings indicate that in addition to being a critical component of the autophagy initiation, Atg1 orchestrates autophagosome elongation via its phosphorylation activity. The data from our study will facilitate future studies on the noncanonical targets of Atg1 and help decipher the Atg1-mediated phosphorylation networks.

**IMPORTANCE** Autophagy-related protein 1 (Atg1) is a serine/threonine protein kinase for autophagy initiation. In contrast to the unicellular yeast, the target proteins phosphorylated by Atg1 are largely unknown in filamentous fungi. In this study, the entomopathogenic fungus Beauveria bassiana was used as a representative of filamentous fungi due to its importance in the applied and fundamental research. We revealed that Atg1 mediates the comprehensive proteome and phosphoproteome, which differ from those revealed in yeast. Further investigation revealed that Atg1 directly phosphorylates the E2-like enzyme Atg3 of the ubiquitin-like conjugation system (ULCS), and the phosphorylation of Atg3 is indispensable for ULCS functionality. Interestingly, the phosphorylation site of Atg3 is conserved among a set of insect- and plant-pathogenic fungi but not in human-pathogenic fungi. This study reveals new regulatory mechanisms of autophagy and provides new insights into the evolutionary diversity of the Atg1 kinase signaling pathways among different pathogenic fungi.

## INTRODUCTION

In eukaryotic cells, autophagy is a vacuole-dependent degradation and turnover mechanism that helps to maintain intracellular homeostasis. The autophagic process may be selective or nonselective, constituting microautophagy or macroautophagy (1). The molecular control of autophagy has mainly been revealed through studies using the model eukaryote Saccharomyces cerevisiae. Following activation of autophagy, target proteins and organelles are wrapped in double-membrane vesicles (autophagosomes) that fuse with vacuoles. The translocated “cargoes” are then degraded by hydrolytic enzymes in the vacuoles for recycling (2). In natural ecosystems, filamentous fungi are characterized by development of robust hyphae and generation of numerous asexual conidia for fungal dispersal and survival (3, 4). Not unexpectedly, autophagy plays a significant role in physiological processes in a wide variety of filamentous fungi (5–7).

Filamentous entomopathogenic fungi (EPF) (e.g., Beauveria bassiana and Metarhizium robertsii) infect insects naturally via cuticle penetration and have been widely developed as mycoinsecticides for biocontrol of insect pests. In addition, the fungi have emerged as model microorganisms for exploring the mechanisms involved in fungal development and fungus-insect interactions (8, 9). Autophagy is associated with the life cycle of entomopathogenic fungi and is critical for a plethora of physiological processes, including asexual development, stress tolerance, and pathogenicity (10). Autophagy involves a series of autophagy-related (*ATG*) genes. Among the 42 known *ATG* genes, 18 represent core *ATG* genes that are conserved among the eukaryotes and are indispensable for autophagic processes. These are categorized into different functional groups and include those that regulate autophagy initiation, vesicle formation, and vesicle maturation, among others (10–12). Atg1 is a serine/threonine protein kinase, and its activity is regulated by Atg13. Once autophagy is induced, Atg13 transforms from a phosphorylated to a dephosphorylated state. The dephosphorylated protein firmly binds to Atg1 to form the autophagy induction complex, which is essential for the nucleation and preautophagosome formation (13).

Aerial conidia are important for the dispersal of EPF in nature and can be used as an active ingredient in practical formulations (14). Atg1, a key Atg in EPF, is crucial for autophagy, and its biological functions in fungal development differ from those of Atg8. During the autophagic process, Atg8 acts as a key hub ubiquitin-like protein in vesicle expansion and autophagosome maturation (12). In B. bassiana, deletion of Atg1 results in approximately 90% reduction in conidial production, but Atg8 contributes to only half of the conidiation capacity (10). In *M. robertsii*, Atg1 is indispensable for conidiation, and Atg8 affects the conidiation process in a nutrient-dependent manner (15). Similar results have been reported for filamentous plant mycopathogens. In Magnaporthe grisea, *ATG1* and *ATG8* have similar effects on fungal conidiation but function differentially in appressorium formation. Atg1 mutants of *M. grisea* (Δ*Mgatg1*) produce very few appressoria (16), and Δ*Mgatg8* mutants form appressoria with normal morphology but without invasive ability (17). In Ustilago maydis, sporidia give birth to daughter cells by budding at or near the tip of the parental cells in liquid culture. Deletion of either *ATG8* or *ATG1* increases the frequency of lateral budding. The Δ*Umatg1* mutants display a higher frequency of cells bearing more than one lateral bud than Δ*Umatg8* mutants (18). These findings indicate that *ATG1* exerts additive influences on the developmental phenotype. As Atg1 is a kinase, we proposed that the key to understanding the additional roles of Atg1 in multiple cellular processes was to comprehensively identify its target substrates. However, besides its autophosphorylation activity (19), the known target proteins and pathways that are regulated by the phosphorylation activity of ATG1 kinase are largely from studies in S. cerevisiae. In yeast, Atg1 phosphorylates a series of proteins, including some components of the autophagy machinery (e.g., Atg9) (20, 21). However, little is known about its biochemical substrates in filamentous fungi.

Isobaric tag for relative and absolute quantification (iTRAQ) analysis has been widely used to identify global proteomes and phosphoproteomes in diverse experimental systems (22). In this study, we performed iTRAQ-based analysis to explore Atg1-dependent proteomics and phosphoproteomics in B. bassiana. The results indicated that Atg1 extensively affects the cellular proteome and phosphoproteome during fungal development. Notably, autophagy-related proteins were also affected by Atg1 kinase, and Atg3 was validated as a downstream target of the Atg1-mediated phosphorylation pathway. Our findings indicate that Atg1 controls fungal physiology by coordinating a complex genetic network.

## RESULTS

### Overview of the Atg1-dependent proteome and phosphoproteome.

**FIG 1:**
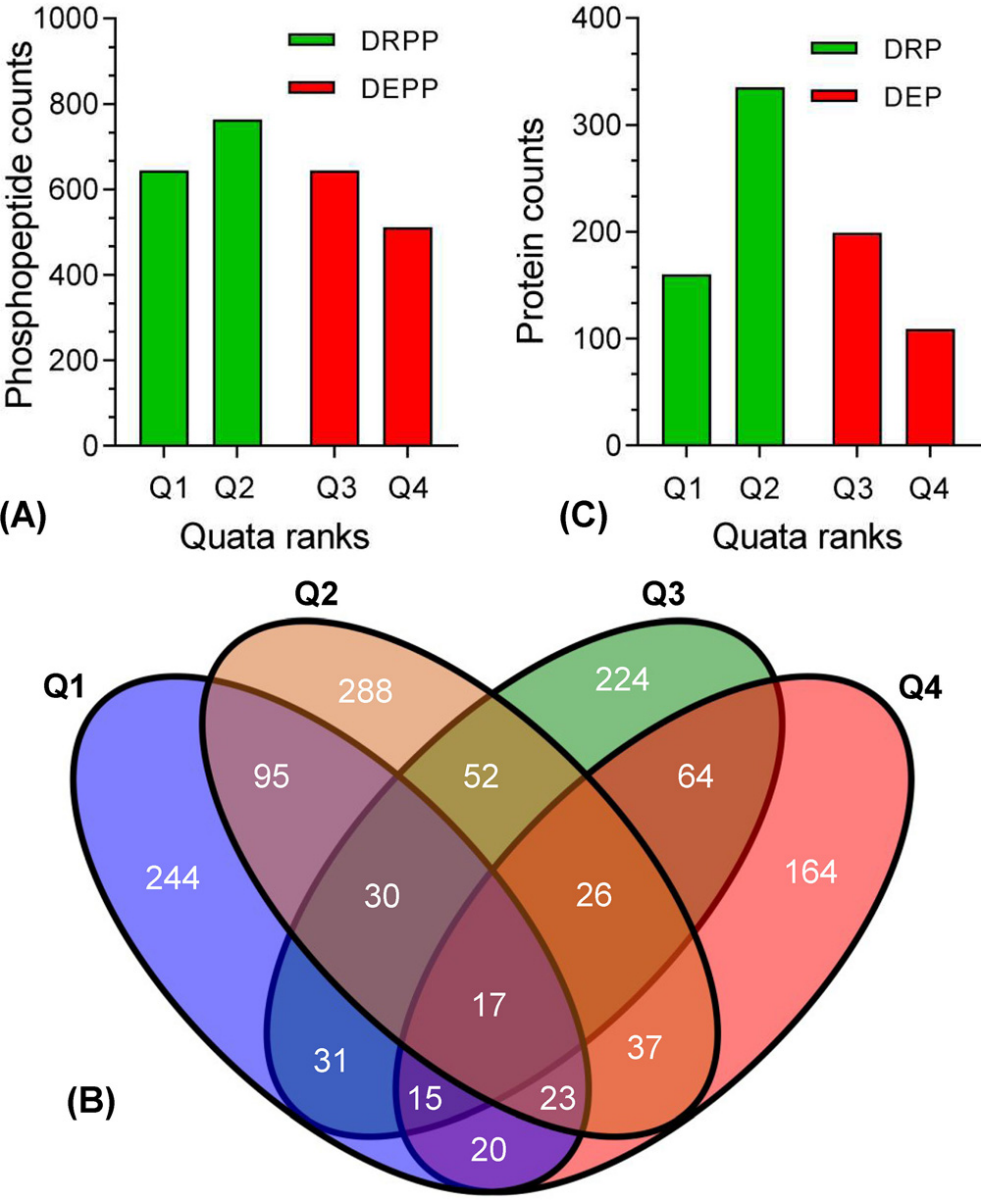
Overview of Atg1-mediated phosphoproteome and proteome in B. bassiana. (A) Ablation of B. bassiana *ATG1* (*BbATG1*) resulted in a plethora of differentially repressed and elevated phosphopeptides (DRPP and DEPP). Based on the fold changes, all differentially altered phosphopeptides (DAPPs) were sorted into four ranks, including Q1 (0 to 0.5), Q2 (0.5 to 0.67), Q3 (1.5 to 2.0), and Q4 (>2.0). DAPPs were mapped on their corresponding proteins. The numbers of proteins with different phosphopeptides (Q1 to Q4) are indicated in a Venn map (B). Loss of *BbATG1* generated a set of differentially repressed and elevated proteins (DRP and DEP) (C).

To determine Atg1-dependent proteomes and phosphoproteins in B. bassiana, global differential protein and phosphoprotein levels between the wild-type (WT) and Δ*Bbatg1* mutant strains were quantified using iTRAQ-based analyses. From these experiments, we identified 10,825 phosphopeptides that mapped to 9,662 unique phosphorylation sites (localization probability > 0.75) on 2,499 quantifiable proteins (Fig. 1A; see also Data Set S1 in the supplemental material). All differentially altered phosphoproteins (DAPPs) were sorted into four ranks in which the site numbers in Q1 were identical to those in Q3 (Fig. 1B). Most (2,153/2,562) of DAPPs were serine (S) residues, followed by threonine residues (383/2,562) and a minimal proportion of tyrosine residues (26/2,562). For the proteome, we identified 5,647 proteins and quantified 4,717 proteins. Ablation of *BbATG1* resulted in 803 differentially altered proteins (DAPs), with 308 elevated and 495 reduced proteins in the mutant strain compared to the WT strain. Rank Q2 contained the maximal number of DAPs (Fig. 1C; Data Set S2).

10.1128/msystems.01463-21.1 DATA SET S1 The differentially altered phosphopeptides in the Δ*Bbatg1* mutant during asexual development in Beauveria bassiana. Download Data Set S1, XLSX file, 0.2 MB. Copyright © 2022 Lin et al. 2022 Lin et al. https://creativecommons.org/licenses/by/4.0/ This content is distributed under the terms of the Creative Commons Attribution 4.0 International license.

10.1128/msystems.01463-21.2 DATA SET S2 The differentially altered proteins in the Δ*Bbatg1* mutant during asexual development in Beauveria bassiana. Download Data Set S2, XLSX file, 0.08 MB. Copyright © 2022 Lin et al. 2022 Lin et al. https://creativecommons.org/licenses/by/4.0/ This content is distributed under the terms of the Creative Commons Attribution 4.0 International license.

### Functional features of the differentially altered phosphoproteomes and proteomes.

**FIG 2:**
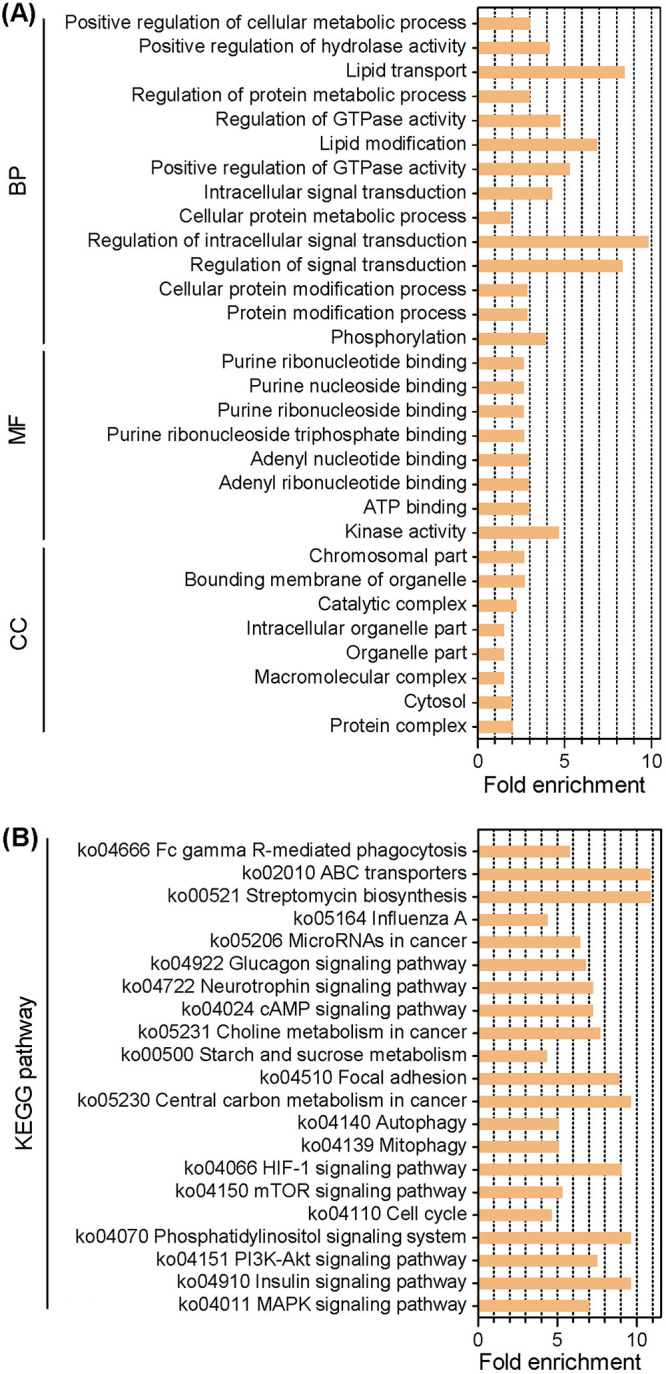
Enrichment analyses for the extremely differentially repressed phosphoproteins in an *ATG1*-null strain of B. bassiana. All differentially altered phosphopeptides were sorted into four ranks. Rank Q1 contained the phosphopeptides with the extremely differentially repressed phosphorylation level (0 to 0.5), and their corresponding proteins were subjected to enrichment analyses with Gene Ontology (A) and KEGG (B) annotation systems.

We next performed an individual enrichment analysis for four ranks (Q1 to Q4) of DAPPs with Gene Ontology (GO) annotation and KEGG pathway analyses. Based on their GO annotations, the DAPPs were enriched in different functional terms belonging to biological process (BP), cellular component (CC), and molecular function (MF) categories. The four ranks displayed significant diversity in functional categories at the secondary level (Data Set S3). Rank Q1 represented DAPPs that were 2-fold reduced in the mutant strain. The category distribution of Q1 at the second level of GO analysis is shown in Fig. 2A. For BPs, the enriched proteins were mainly associated with lipid metabolism (e.g., lipid transport [GO: 0006869] and lipid modification [GO: 0030258]), regulation of intracellular signal transduction (GO: 1902531), and regulation of GTPase activity (GO: 0043087). For MFs, most categories (7/8) were involved in nucleoside/nucleotide binding (e.g., ATP binding [GO: 0005524]). For CCs, the three largest categories were macromolecular complex (GO: 0032991), organelle part (GO: 0044422), and intracellular organelle part (GO: 0044446). Using the KEGG categorization system, the DAPPs were overrepresented in different pathways, and the four ranks showed high complexity in functional pathways (Data Set S3). As illustrated in Fig. 2B, the enriched pathways for rank Q1 were mainly associated with signal transduction (e.g., mitogen-activated protein kinase [MAPK] signaling pathway), metabolism (e.g., starch and sucrose metabolism), cell cycle, and autophagy. Notably, autophagy-related proteins included Atg2, Atg3, Atg9, and Atg11. Rank Q4 included DAPPs that were 2-fold elevated in Δ*Bbatg1* mutant strain. As for the BP category, the enriched categories were largely associated with phosphorus metabolism (e.g., phosphorus metabolic process [GO: 0006793]) and protein modification (e.g., cellular protein modification process [GO: 0006464]). For MFs, four categories were involved in molecular binding (e.g., vitamin binding [GO: 0019842]), two categories were related to transferase activity (e.g., phosphotransferase activity (GO: 0016773), and two categories involved kinase (e.g., protein kinase activity [GO: 0004672]). For CCs, the largest category was related to the microtubule cytoskeleton (GO: 0015630). According to KEGG classification, the DAPPs of Q4 were enriched with the AMP-activated protein kinase (AMPK) signaling pathway and autophagy among others. Autophagy-related proteins included Atg11 and Atg13.

10.1128/msystems.01463-21.3 DATA SET S3 Enrichment analyses for four ranks of differentially changed proteins in the *ATG1*-null strain of Beauveria bassiana. Download Data Set S3, XLSX file, 0.03 MB. Copyright © 2022 Lin et al. 2022 Lin et al. https://creativecommons.org/licenses/by/4.0/ This content is distributed under the terms of the Creative Commons Attribution 4.0 International license.

**FIG 3:**
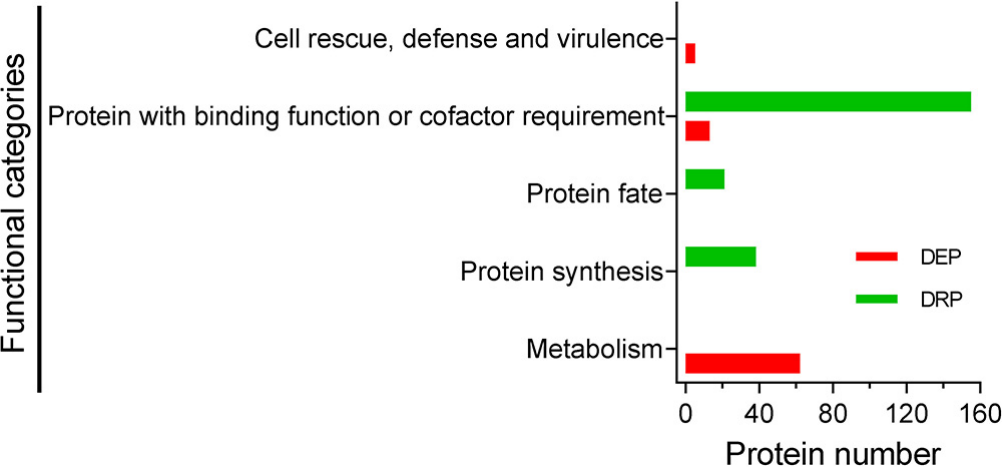
Functional distribution analysis of the Atg1-mediated proteome in B. bassiana. Differentially altered proteins (DAPs) were determined by comparative proteomics between the wild-type and Δ*Bbatg1* mutant strains. FunCat algorithm was used to individually sort the differentially repressed and elevated proteins (DRP and DEP), and these DAPs are represented in five functional categories.

As for the ATG1-mediated proteome, FunCat analysis showed that the differentially reduced proteins (DRPs) were enriched in the categories associated with protein synthesis, protein fate, and protein with binding function (Fig. 3; Data Set S4). The DRPs involved in protein synthesis included several ribosomal proteins (e.g., 60S ribosomal protein L11), those involved in protein fate included many proteases/peptidases (e.g., peptidase family M3) and proteasome subunits (e.g., 26S proteasome regulatory subunit RPN5), and those involved in protein with binding function included protein-binding proteins (e.g., ASF1-like histone chaperone) and RNA-binding proteins (e.g., RNA recognition domain-containing protein). For the differentially elevated proteins (DEPs) (Fig. 3; Data Set S4), the proteins were overrepresented in the following: (i) metabolism, including a series of enzymes crucial for biosynthesis of phenylalanine (e.g., aspartate aminotransferase) and carbohydrate metabolism (e.g., beta-1,3-exoglucanase); (ii) protein with binding function, including many NAD/NADP-binding proteins (e.g., alcohol dehydrogenase); and (iii) cell rescue, defense, and virulence, including pectin lyase and LysM domain-containing protein, among others.

10.1128/msystems.01463-21.4 DATA SET S4 Functional sortation of differentially altered proteins in the Δ*Bbatg1* mutant of Beauveria bassiana during development. Download Data Set S4, XLSX file, 0.01 MB. Copyright © 2022 Lin et al. 2022 Lin et al. https://creativecommons.org/licenses/by/4.0/ This content is distributed under the terms of the Creative Commons Attribution 4.0 International license.

### Analysis of phosphorylation motifs.

**FIG 4:**
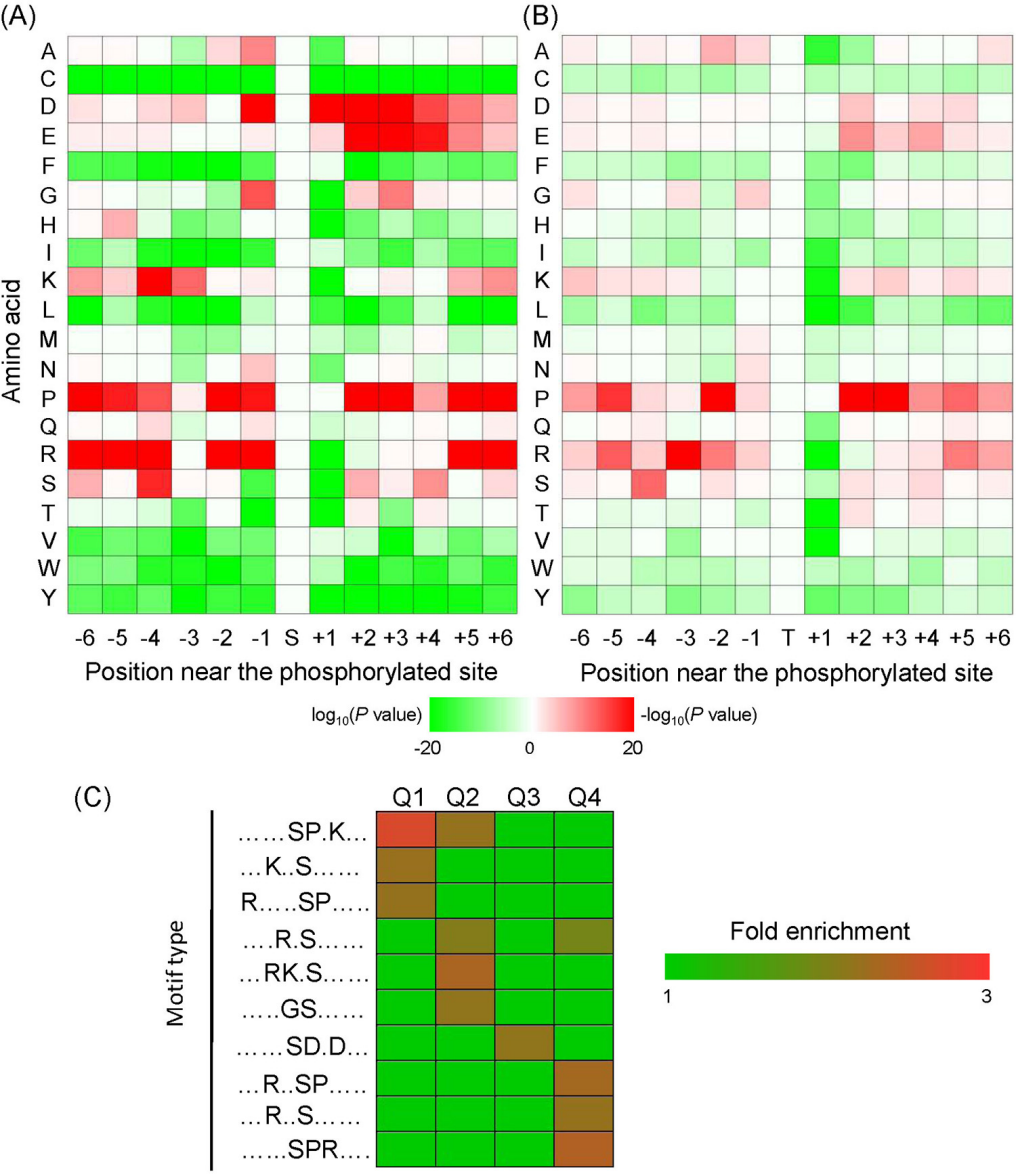
Motif analyses of the phosphorylated peptides. Phosphorylation motifs were constructed using Motif-X software. Heat maps indicate the frequencies of the amino acid residues around the phosphorylation sites of serine (A) and threonine (B). (C) Changes in phosphorylation levels of phosphopeptides were divided into four ranks, including Q1 (0 to 0.5), Q2 (0.5 to 0.67), Q3 (1.5 to 2.0), and Q4 (>2.0). The heat map shows the enriched motifs in each rank.

To explore the ATG1-dependent phosphoregulation of protein substrates, sequences of the phosphorylated peptides were submitted to the Motif-X program. Based on Motif-X analysis, 33 serine motifs and 5 threonine motifs were identified. Motif 35 (RXXTP) (“X” represents a random amino acid residue) displayed a maximal fold increase (30.52), and motif 33 (SXXXS) was enriched only 1.69-fold (Table 1). A survey of these motifs indicated that amino acid residues surrounding the phosphorylation sites displayed different conserved properties (Fig. 4A and B). Among these two types of motifs, proline (P) and arginine (R) showed similar enrichment modes, but these amino acids displayed a higher frequency in the serine motifs. Among serine motifs, aspartate (D) was conserved at position −1 and positions 1 to 4, whereas glutamate (E) typically appeared at positions 2 to 4. Additionally, lysine (K) was conserved at position −4 in the serine motifs.

**TABLE 1:** Phosphorylation motifs identified in the *B.* bassiana Atg1-mediated phosphoproteome

Serial no.	Motif	Motif score	Foreground		Background		Fold increase
			Matches	Size^*a*^	Matches	Size^*a*^	
1	….P.SP…..	32	501	7,861	2,542	402,022	10.08
2	…R..SP…..	32	396	7,360	1,691	399,480	12.71
3	……SP.P…	31.65	265	6,964	1,991	397,789	7.6
4	…RR.S……	32	484	6,699	2,532	395,798	11.29
5	……SP.K…	32	150	6,215	945	393,266	10.04
6	……SP.R…	32	182	6,065	1,348	392,321	8.73
7	.R.R..S……	32	212	5,883	1,973	390,973	7.14
8	……SPR….	24.12	110	5,671	954	389,000	7.91
9	R…..SP…..	22.92	101	5,561	946	388,046	7.45
10	…RK.S……	32	119	5,460	1,042	387,100	8.1
11	……SP…P.	22.29	119	5,341	1,268	386,058	6.78
12	.H.R..S……	32	100	5,222	834	384,790	8.84
13	……SP…..	16	775	5,122	13,844	383,956	4.2
14	…RP.S……	27	116	4,347	1,500	370,112	6.58
15	…KR.S……	32	116	4,231	1,165	368,612	8.67
16	……S.DE…	32	167	4,115	1,873	367,447	7.96
17	..RR..S……	26.55	101	3,948	1,386	365,574	6.75
18	R..S..S……	32	145	3,847	2,958	364,188	4.64
19	….R.S……	16	541	3,702	18,764	361,230	2.81
20	…RS.S……	25.87	117	3,161	1,946	342,466	6.51
21	……SD.D…	32	123	3,044	1,821	340,520	7.56
22	…R..S……	16	395	2,921	13,485	338,699	3.4
23	……SD.E…	32	113	2,526	1,381	325,214	10.53
24	……S..G…	16	321	2,413	23,401	323,833	1.84
25	……S..D…	16	280	2,092	18,357	300,432	2.19
26	……S….P.	16	236	1,812	18,017	282,075	2.04
27	……S..E…	16	188	1,576	14,812	264,058	2.13
28	……SD…..	16	174	1,388	13,154	249,246	2.38
29	…S..S……	16	226	1,214	25,051	236,092	1.75
30	…K..S……	16	123	988	10,524	211,041	2.5
31	…..GS……	12.83	126	865	14,600	200,517	2
32	…D..S……	9.08	101	739	13,448	185,917	1.89
33	……S…S..	7.36	109	638	17,410	172,469	1.69
34	….P.TP…..	32	211	1,427	2,140	295,789	20.44
35	…R..TP…..	32	148	1,216	1,171	293,649	30.52
36	……TPP….	32	124	1,068	1,348	292,478	25.19
37	……TP…..	16	467	944	16,162	291,130	8.91
38	…R..T……	16	160	477	16,343	274,968	5.64

a Number of peptides.

Enrichment analysis indicated that the identified motifs exhibited different distributions over the four ranks of DAPPs (Fig. 4C; Data Set S5). Both ranks Q2 and Q4 had four enriched motifs, and rank Q1 had three enriched motifs. Only one motif (motif 21 [SDXD]) was significantly overrepresented in rank Q3. Both Q1 and Q2 had motif 5 (SPXK), and motif 19 (RXS) was enriched in Q2 and Q4. The four ranks had no overlapping enrichment.

10.1128/msystems.01463-21.5 DATA SET S5 Enrichment analyses for the motifs in the different ranks of differentially altered phosphopeptides. Download Data Set S5, XLSX file, 0.01 MB. Copyright © 2022 Lin et al. 2022 Lin et al. https://creativecommons.org/licenses/by/4.0/ This content is distributed under the terms of the Creative Commons Attribution 4.0 International license.

### Detecting Atg1 targets.

In yeast Atg1 targets, the phosphorylation acceptor serine is characteristic of that leucine (L), methionine (M) and serine presented at the position −3 (20). On the basis of this information, a total of 183 potential targets were predicted in B. bassiana (Data Set S6). Protein counts for L, M, and S residues were 60, 13, and 110, respectively. A protein (locus tag BBA_04421, with serine at the position −3) was homologous to Pax, which is an Atg1 substrate in Drosophila melanogaster (23), but the consensus sequence for phosphorylation site was not seen in D. melanogaster Pax. No other B. bassiana targets had homologs in the known Atg1 substrates revealed for yeast, fruit flies, and humans (20, 23, 24).

10.1128/msystems.01463-21.6 DATA SET S6 Potential BbAtg1 substrates and their homologs in yeast, fruit flies, and humans. Download Data Set S6, XLSX file, 0.02 MB. Copyright © 2022 Lin et al. 2022 Lin et al. https://creativecommons.org/licenses/by/4.0/ This content is distributed under the terms of the Creative Commons Attribution 4.0 International license.

Atg1 functions as a protein kinase and phosphorylates downstream targets (20). Proteins with repressed phosphorylation levels and without changes in protein levels represent likely targets of Atg1 kinase activity in B. bassiana. We first screened 577 phosphorylated sites (in 335 proteins) that showed a significant change in phosphorylation, without significant change in their protein levels (Data Set S7). We found that 165 proteins contained only repressed phosphorylation sites, whereas 117 contained only elevated phosphorylation sites. Further, 53 proteins contained both repressed and elevated phosphorylation sites.

10.1128/msystems.01463-21.7 DATA SET S7 Phosphopeptides regulated only by the phosphorylation activity of Atg1 in Beauveria bassiana. Download Data Set S7, XLSX file, 0.1 MB. Copyright © 2022 Lin et al. 2022 Lin et al. https://creativecommons.org/licenses/by/4.0/ This content is distributed under the terms of the Creative Commons Attribution 4.0 International license.

Taken together, BbAtg1 showed potential kinase activity on autophagy-related proteins (i.e., BbAtg2, BbAtg3, and BbAtg11) (Data Set S7). Atg3 acts as an E2-like enzyme in the ubiquitin-like conjugation system (ULCS), which is essential for autophagy (11). In BbAtg3, two adjacent serine residues displayed repressed phosphorylation levels with different localization probabilities (Data Set S1), and their phosphorylation mode was not included in the identified motifs. Therefore, Atg3 (locus tag BBA_07719) was chosen as a representative target to validate the role of Atg1 in phosphoregulation of autophagy.

**FIG 5:**
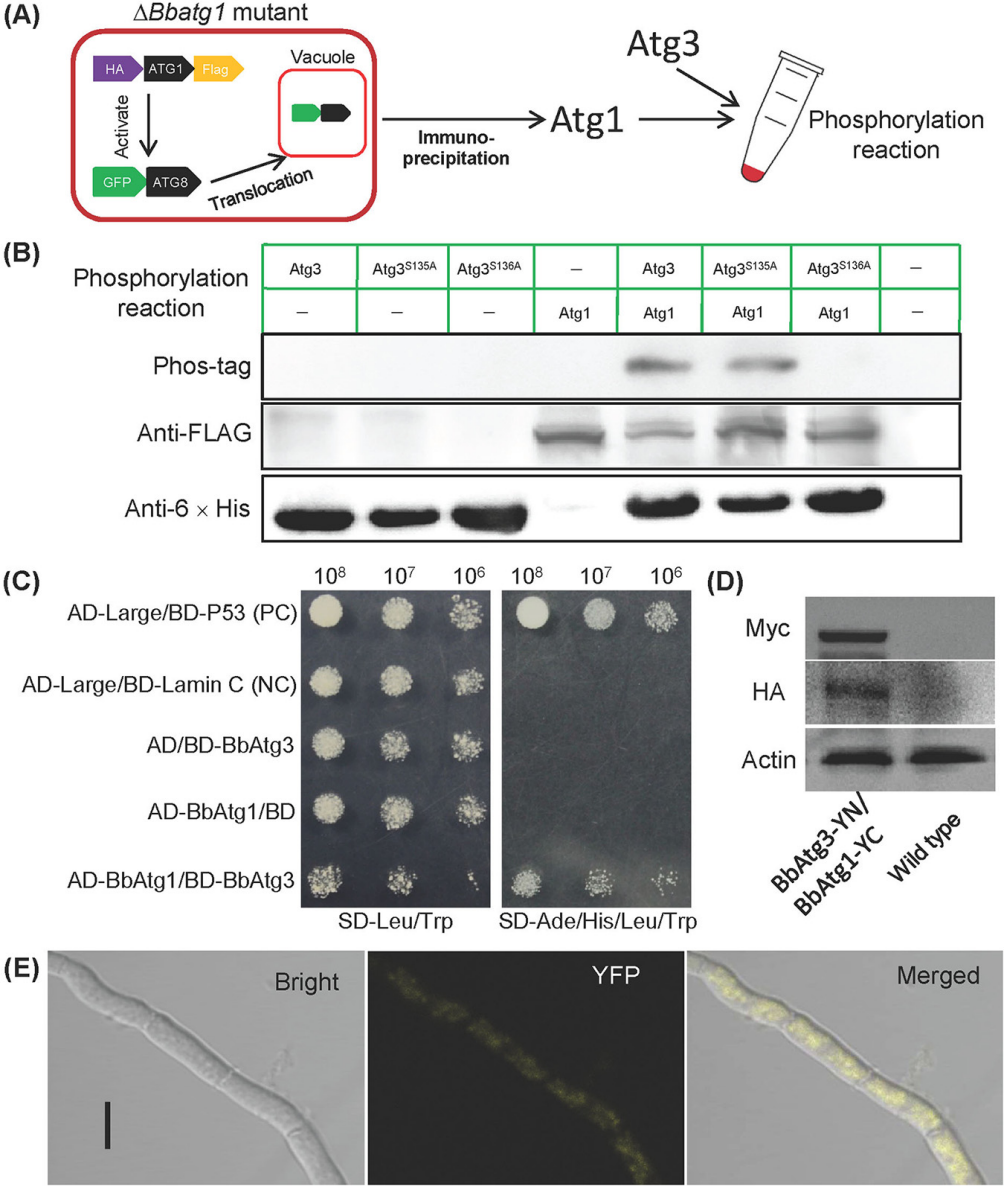
Assay for direct phosphorylation of BbAtg1 on BbAtg3. (A) The human influenza virus hemagglutinin (HA) and Flag gene were fused to the 5′ and 3′ termini of *BbATG1*, and the resulting gene was transformed into the Δ*BbAtg1* mutant strain with the autophagy reporter gene (*GFP-ATG8*). The functionality was indicated by the translocation of GFP-Atg8 into vacuoles. The resultant proteins were isolated and purified by immunoprecipitation. In a kinase assay, BbAtg1 was incubated with BbAtg3 and its mutant forms, and then the phosphorylated products were resolved on an SDS-PAGE gel and detected by Western blotting (B). (C) Y2H test. The yeast transformant with BbAtg1 and BbAtg3 grew well on the SD/-Leu-Trp-Ade-His medium. Positive (PC) and negative (NC) controls were provided with the kit. BiFC assay was used to detect the *in vivo* protein interaction. *BbATG1-YC* and *BbATG3-YN* were introduced into the wild-type strain, and the resultant transformants were cultured on SDAY in SDB for 3 days. Their products in mycelia were determined by immunoblotting (D), and the fluorescent signals were examined under a laser confocal microscope (E). Bar: 5 μm.

To determine whether BbAtg1 directly phosphorylates BbAtg3, BbAtg1 was endogenously prepared in the Δ*Bbatg1* mutant strain (Fig. 5A; Fig. S1A), and its functionality was judged by Atg8 translocation from the cytoplasm into vacuoles (Fig. S1B). In the phosphorylation assay, BbAtg1 was incubated with BbAtg3 and its mutants, which were prepared in a bacterial expression system (Fig. S1C). The resulting phosphorylated proteins were analyzed through immunoblot assay (Fig. 5B). The phosphorylation events were observed in the wild-type BbAtg3 and its derivative with mutation at position S135, and mutation of S136 resulted in the absence of BbAtg3 phosphorylation. In a yeast two-hybrid (Y2H) assay (Fig. 5C), only the yeast transformants with *BbATG1* and *BbATG3* grew well on the selection medium, which was identical to the strain as the positive control. In a bimolecular fluorescence complementation (BiFC) assay, *BbATG3* and *BbATG1* were fused to the N and C termini of the split yellow fluorescent protein (YFP) gene, respectively. Both fusion genes were successfully expressed in B. bassiana (Fig. 5D), and significant fluorescent signals were observed in mycelial cytoplasm (Fig. 5E). These results indicated BbAtg3 acts as the direct target of BbAtg1, and its position S136 is a critical phosphorylation site.

10.1128/msystems.01463-21.8 FIG S1 Preparation of BbAtg1 and BbAtg3. The tagged-BbATG1 was transformed into the Δ*Bbatg1* mutant strain with the hybrid gene of *GFP-ATG8*. The resultant transformant was cultured in SDB medium. (A) Presence of BbAtg1 in cell lysate was analyzed by Western blotting. Actin was used as an endogenous reference. (B) Assay for *in vivo* functionality of BbAtg1. The 2-day-old submerged mycelia were stressed under starvation. The functionality of BbAtg1 was indicated by the translocation of GFP-Atg8 into vacuoles (stained with FM4-64). Scale bar: 5 μm. (C) Preparation of BbAtg3. The predicted phosphorylation sites S135 and S136 of the wild-type BbAtg3 (WA) were mutated into alanine, generating two mutant forms, MA1 and MA2, respectively. Three genes were expressed in a bacterial expression system. The resultant proteins were purified from cell lysate with Ni-chelating affinity chromatography and analyzed on SDS-PAGE. Lane M, protein molecular marker. Download FIG S1, TIF file, 2.3 MB. Copyright © 2022 Lin et al. 2022 Lin et al. https://creativecommons.org/licenses/by/4.0/ This content is distributed under the terms of the Creative Commons Attribution 4.0 International license.

On the basis of sequence alignment analyses (Fig. S2A), the homologs of Atg3 from filamentous fungi were sorted into a group, and clustering by fungal lifestyle, i.e., saprobes and plant, insect, or human pathogens, was not significantly observed. BbAtg3 had higher similarity to Atg3 of Cordyceps militaris than to those of two Metarhizium species. The phosphorylation acceptor of BbAtg3 was serine, and leucine localized at position −3 of the phosphosite. This character was conserved in the homologs of the insect- and plant-pathogenic fungi and not found in the homologs from two Aspergillus species, yeast, plant, or animal (Fig. S2B). In addition, Neurospora crassa Atg3 had methionine at the position −3 of the phosphorylation acceptor (serine).

10.1128/msystems.01463-21.9 FIG S2 Bioinformatic and functional analyses of BbAtg3. (A) Relationships among the Atg3 homologs were revealed through neighbor joining analysis, and the bootstrap values >50% from 1,000 replicates are shown at the supported node. Abbreviations for organism species plus GenBank accession numbers are shown. Afu, Aspergillus fumigatus; And, Aspergillus nidulans; Ath, Arabidopsis thaliana; Bba, Beauveria bassiana; Cal, Candida albicans; Cel, Caenorhabditis elegans; Cmi, Cordyceps militaris; Dme, Drosophila melanogaster; Fgr, Fusarium graminearum; Hsa, Homo sapiens; Mac, Metarhizium acridum; Mgi, Magnaporthe grisea; Mro, Metarhizium robertsii; Nc, Neurospora crassa; Sce, Saccharomyces cerevisiae. (B) Alignment analysis of the phosphorylation sites. Red frame indicates the serine acceptor of phosphorylation. Blue frame indicates the amino acid residue at the position −3. (C) Target replacement was accomplished via homologous recombination with disruption vector. (D) PCR confirmation of recombination events in the gene disruption and complementation mutant strains. (E) Southern blotting. Genomic DNAs were digested and probed by chemiluminescent method. The sizes of the DNA fragments are indicated at the left side of the image. Download FIG S2, TIF file, 3.6 MB. Copyright © 2022 Lin et al. 2022 Lin et al. https://creativecommons.org/licenses/by/4.0/ This content is distributed under the terms of the Creative Commons Attribution 4.0 International license.

### Atg3 is required for autophagy during stress response and conidiation in *B.* bassiana.

Physiological functions of *BbATG3* were examined by constructing gene disruption and complementation mutants (Fig. S2C). All genes with recombination and integration events were successfully verified by PCR and Southern blot analysis (Fig. S2D and E).

**FIG 6:**
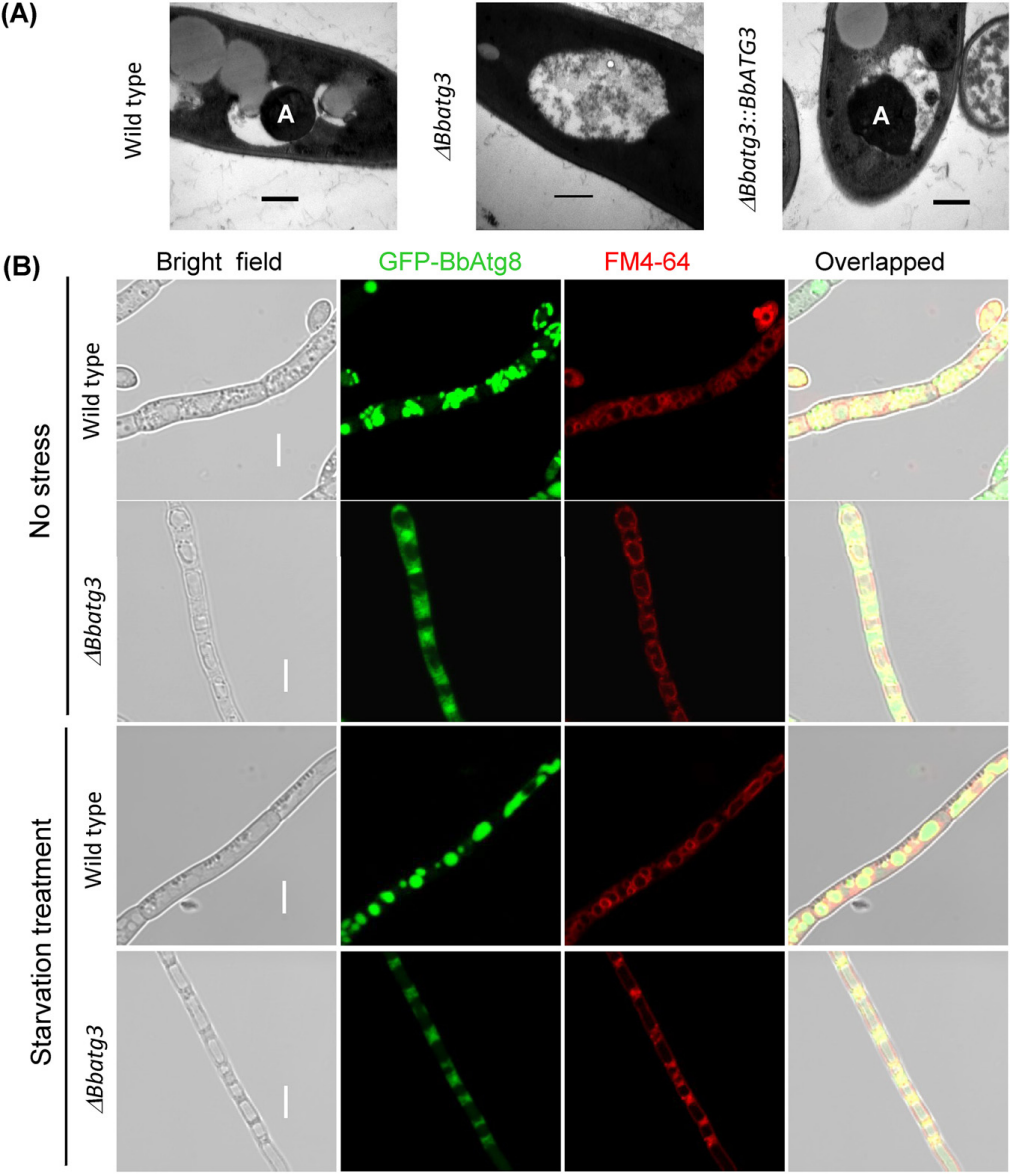
*BbATG3* is required for autophagy in B. bassiana under starvation. (A) Representative images of the autophagic process. Conidia of the indicated strain were inoculated into SDB, and the resultant mycelia were stressed under starvation in mineral solution for 3 h. Autophagic bodies (indicated with “A”) in mycelial vacuoles were examined by transmission electronic microscopy. Scale bars represent 0.2 μm. (B) Fluorescent protein indicating the autophagic process. A hybrid gene, *ATG8*-*GFP*, was transformed into the wild-type and Δ*Bbatg3* mutant strains, and the protein products show the trafficking of autophagosomes/autophagic bodies. Ablation of *BbATG3* interrupted the translocation of autophagosome from cytoplasm to vacuoles. Scale bars indicate 10 μm.

**FIG 7:**
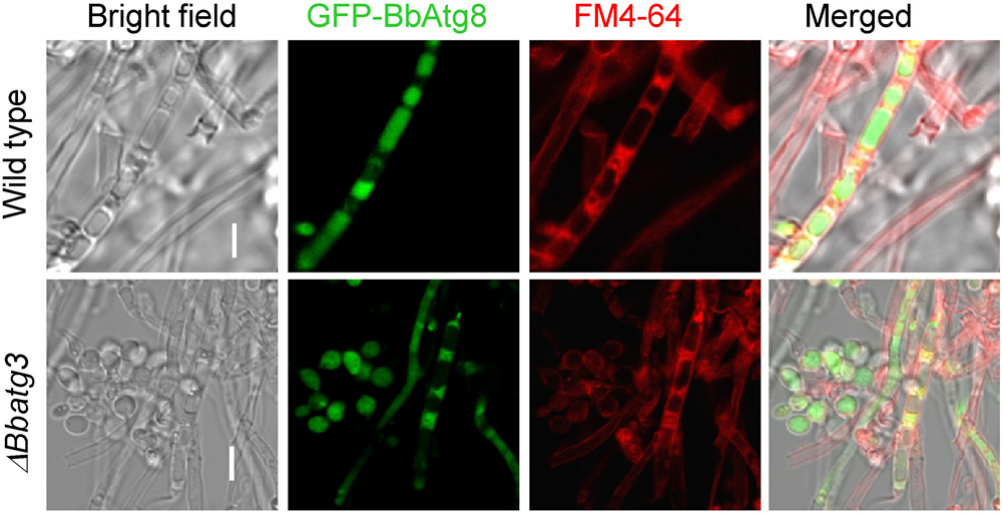
*BbATG3* is necessary for B. bassiana autophagy during aerial development. The genetically modified strains were constructed as shown in Fig. 5B. The indicated strains were grown on SDAY plates for 3 days at 25°C. In the wild-type strain, significant green signals were observed in mycelial vacuoles; however, no green signal was translocated into the vacuoles in Δ*Bbatg3* mutant strain. Scale bars represent 5 μm.

After subjecting the cells to stress under starvation conditions for 3 h, autophagic bodies were clearly observed in the mycelia of the WT and complemented strains, and a few were also detected in the Δ*Bbatg3* strain (Fig. 6A). These results agreed with those obtained with the mutants lacking other ULCS genes (e.g., *ATG5*, *ATG7*, and *ATG8*) (10, 25, 26). Atg8-green fluorescent protein (GFP) fusion protein revealed autophagosomes and autophagic bodies in fungal mycelia (Fig. 6B). Strong GFP signals were observed in the vesicles of the WT strain and remained in the cytoplasm in the Δ*Bbatg3* mutant strain. Starvation stress caused the GFP signals to translocate into vacuoles in the WT strain. However, in the Δ*Bbatg3* mutant, the GFP signals were not significantly present in the vacuoles. When cultured on Sabouraud dextrose agar-yeast extract (SDAY) plates (Fig. 7), GFP signals filled the vacuoles in the WT strain, but not in the Δ*Bbatg3* mutant strain. These results revealed that *BbATG3* is required for the formation of autophagic bodies in B. bassiana.

### Validation of the phosphorylation sites in *B.* bassiana Atg3.

**FIG 8:**
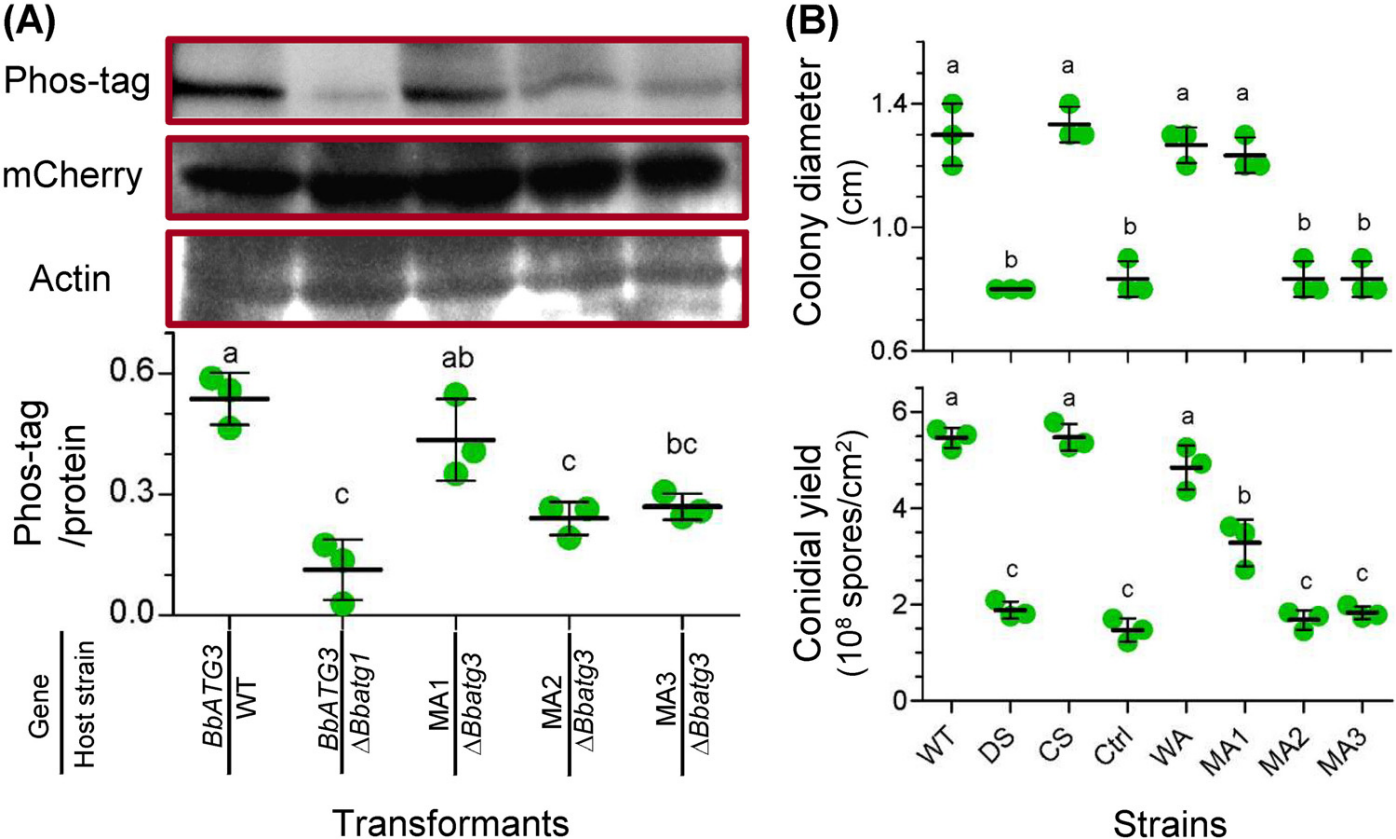
Biochemical and physiological roles of the phosphorylation sites of B. bassiana ATG3. (A) To test the requirement of Atg1 for the Atg3 phosphorylation, *BbATG3* was transformed into the WT and Δ*Bbatg1* mutant strains. To determine the phosphorylation sites in BbAtg3, two serine residues (S135 and S136) was mutated individually and simultaneously. The resultant genes with single mutations at S135 and S136 were designated MA1 and MA2, respectively, and the gene with double mutation was named as MA3. The mutated gene was fused to *mCherry* gene and transformed into the Δ*Bbatg3* mutant strain. (Top) Protein sample was resolved by SDS-PAGE and transferred onto a PVDF membrane. The phosphorylation signals were detected with the Phos-tag staining method. The mCherry protein level was determined using Western blotting, which also showed the BbAtg3 level. Actin was used as a reference for protein samples. (Bottom) The band intensity of Phos-tag and protein staining was determined, and their relative value was used to evaluate the phosphorylation level. (B) Roles of phosphorylation sites in fungal tolerance of oxidative stress (top) and conidiation (bottom). The assay was first performed in the WT, Δ*Bbatg3* (DS), and complementation (CS) strains. To test the roles of the phosphorylation sites, mutated genes (MA1, MA2, and MA3) were integrated into the Δ*Bbatg3* mutant strain. Additionally, the wild-type ATG3 (WA) and *mCherry* gene (Ctrl) were used as positive and negative controls, respectively. Oxidative stress was established by including menadione (0.02 mM) in CZA medium. Colony diameter was measured at 7 days postincubation. Conidial production was examined by incubating fungal strains on SDAY plates for 7 days. Lowercase letters indicate a significant difference between the different strains (Tukey’s honestly significant difference [HSD]; *P* < 0.05). Error bars: standard deviation.

To explore the kinase effects of BbAtg1 on BbAtg3, we compared the phosphorylation levels of BbAtg3 in the WT and Δ*Bbatg1* mutant strains. As shown in Fig. 8A, the phosphorylation signals corresponding to BbAtg3 were significantly reduced in Δ*Bbatg1* mutant strain compared to that in the WT strain. BbAtg3 contains two serine phosphorylation sites at positions 135 and 136 that are affected by BbAtg1. Therefore, we generated three *BbATG3* mutants with single and double mutations at the phosphorylation sites. Mutation of S135 (MA1) did not significantly reduce phosphorylation. However, mutation of S136 (MA2) resulted in a significant decrease in the phosphorylation signals, similar to that of BbAtg3 in the Δ*Bbatg1* mutant strain. The double mutant (MA3) displayed phosphorylation intensity similar to that of MA2. These results are supported by the data in Fig. 5B.

Ablation of *BbATG3* resulted in a significant decrease in colony diameter under menadione stress (Fig. 8B, top). Compared to the WT and complementation mutant strains, the Δ*Bbatg3* strain exhibited a marked decrease in conidial production on plates, with approximately 65% reduction at 8 days postincubation (dpi) (Fig. 8B, bottom). These data indicate that *BbATG3* contributes to fungal aerial sporulation and stress response to menadione stress. To identify the role of the phosphorylation sites in fungal physiology, we complemented the Δ*Bbatg3* mutant strain with different mutated *BbATG3* genes (Fig. 8B, top). Under oxidative stress, *BbATG3* with the S135 mutation restored the colony size of the Δ*Bbatg3* mutant strain. However, *BbATG3* with the S136 mutation, including single and double mutations, could not restore the colony size of the Δ*Bbatg3* mutant strain. The control strain (expressing only *mCherr*y) did not mediate any recovery effect. These data indicate that site S136 is critical for fungal resistance to menadione stress. As for conidiation (Fig. 8B, bottom), neither MA2 nor MA3 could restore the conidial yield in the Δ*Bbatg3* mutant strain. The conidial yields of the MA2 and MA3 strains were similar to those of the control and Δ*Bbatg3* mutant strains. MA1 only partially restored the conidial yield in the Δ*Bbatg3* mutant. These results indicate that mutations in the two phosphorylation sites have different recovery effects on conidial yield in the Δ*Bbatg3* mutant strain.

**FIG 9:**
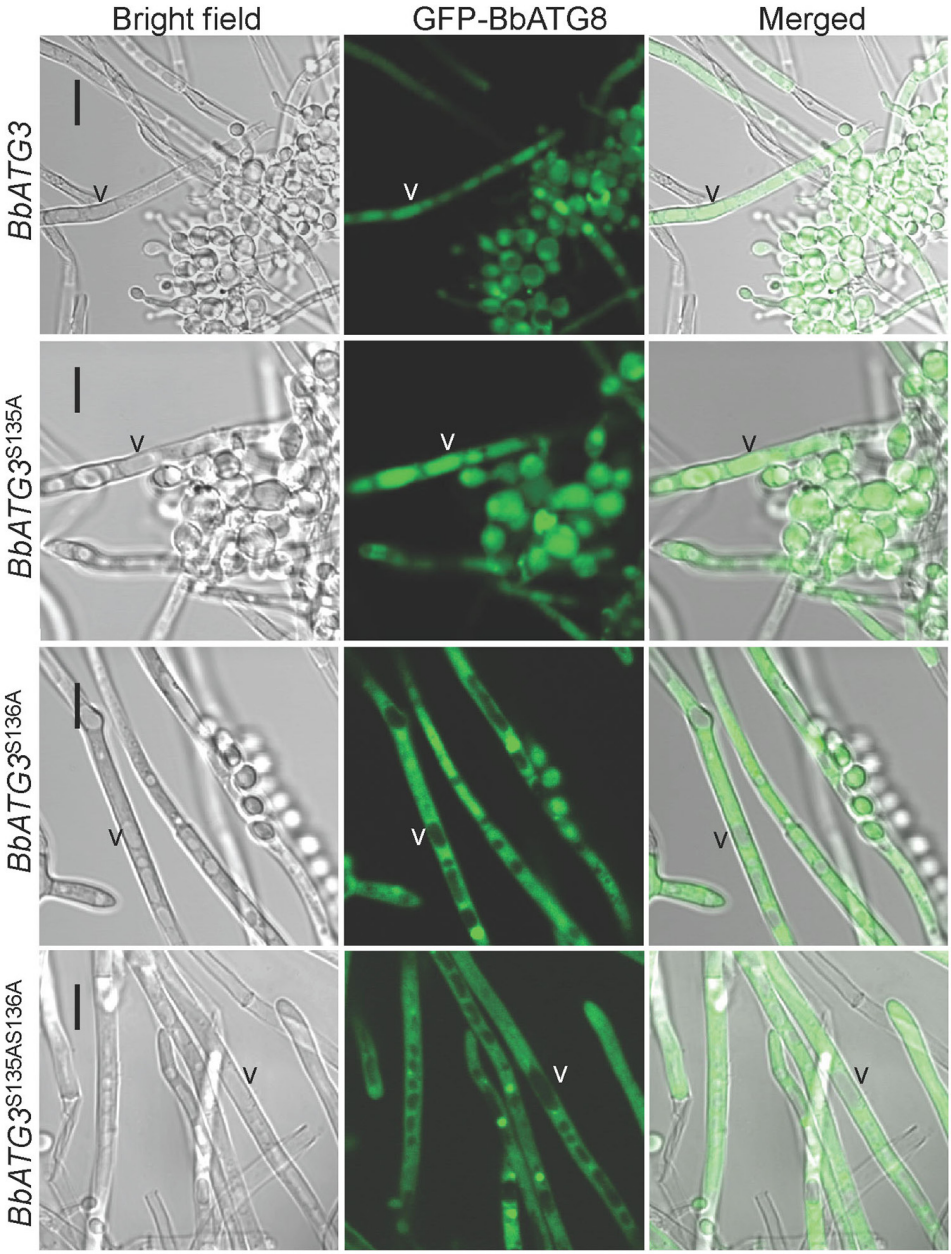
Requirements of the BbAtg3 phosphorylation sites for autophagic flux during *B.* bassiana development. To determine the necessity of two predicted phosphorylation sites in BbAtg3, the two serine residues (S135 and S136) was mutated into alanine (A) individually and simultaneously. The resultant gene was separately introduced into the A3-GA8 strain, in which autophagy was indicated with GFP-Atg8 fusion protein. Wild-type *BbATG3* restored autophagic flux in vacuoles of the Δ*Bbatg3* strain. Mutation at S135 did not affect the recovery effects of *BbATG3*. However, mutation of S136, including single and double mutations together with S135, disrupted the recovery effects of *BbATG3*. “V” indicates the vacuoles in fungal mycelia. Scale bar: 10 μm.

We also analyzed the restoration of autophagic flux (visualized using GFP-Atg8) in the Δ*Bbatg3* mutant by various mutated *BbATG3* genes (Fig. 9). On SDAY plates, many vacuoles formed in the mycelia of the Δ*Bbatg3* mutant strain. When wild-type *BbATG3* (WA) was introduced into the Δ*Bbatg3* mutant, green signals were clearly observed in the vacuoles. MA1 also caused the translocation of green signals into vacuoles. However, the translocation of the green signals into the vacuoles was significantly blocked in the Δ*Bbatg3* strain expressing MA2 or MA3. These experiments indicated that the phosphorylation site S136 is required for autophagy.

**FIG 10:**
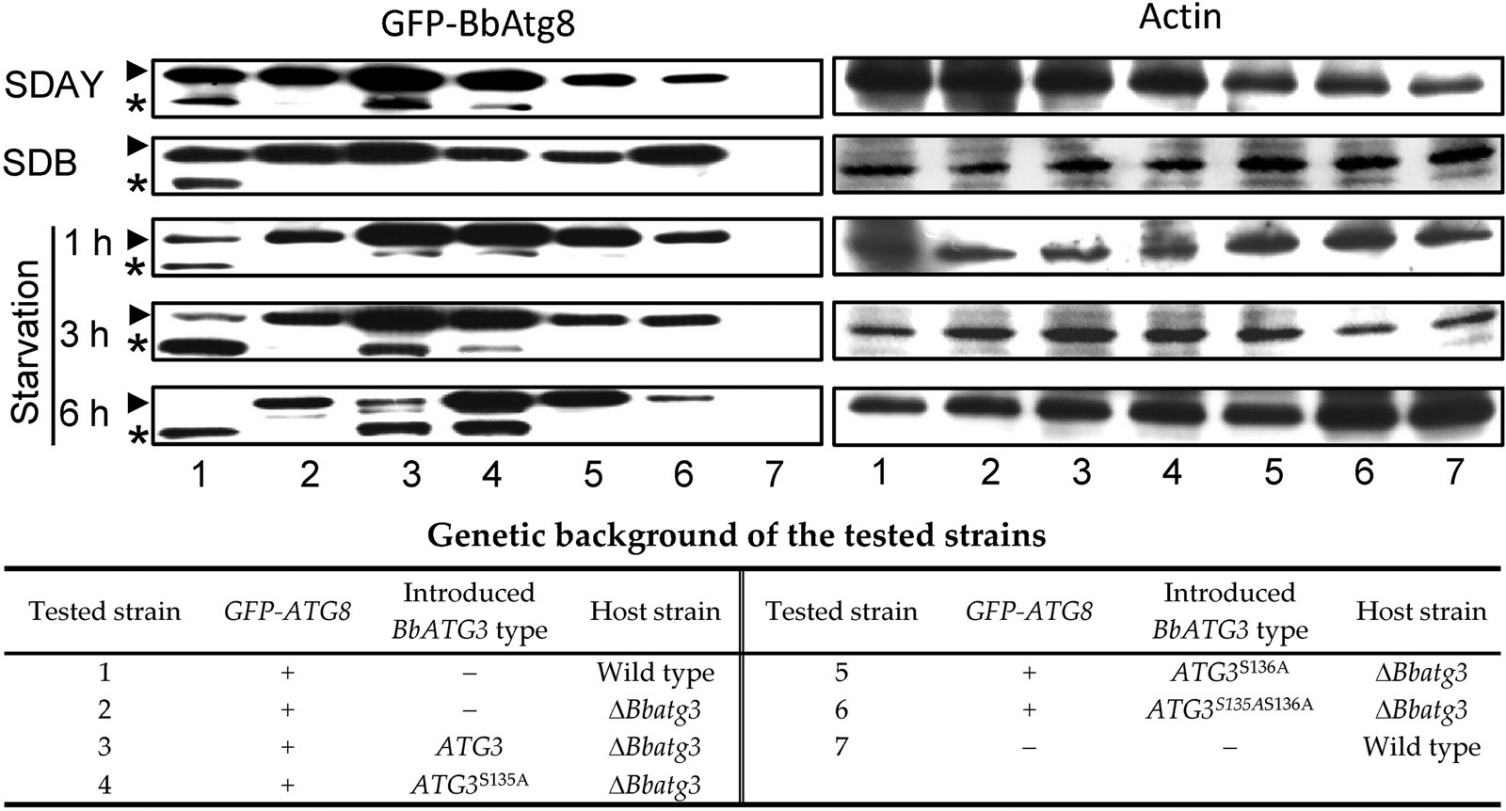
Assay for Atg8 lipidation. To determine the requirements of phosphorylation sites for Atg8 lipidation, *BbATG3* and its mutants were transformed into the Δ*Bbatg3* mutant strain with the *GFP*-tagged *ATG8*. For aerial and submerged mycelia, fungal strains were cultured on SDAY for 5 days and in SDB for 2 days, respectively. For starvation stress, the submerged mycelia were incubated in mineral solution for 1, 3, and 6 h. The cell lysate was resolved on an SDS-PAGE gel with urea, and GFP-Atg8 was probed with anti-GFP antibody. Protein quality in the sample was controlled by actin. Arrowheads and asterisks indicate the GFP-Atg8 and its phosphatidylethanolamine-tailed form. “+” and “−” indicate presence and absence of the indicated genetic factor.

Additionally, we analyzed the requirement of the phosphorylation site for Atg8 lipidation (Fig. 10). On SDAY plates, the conjugation of Atg8 to phosphatidylethanolamine (PE) was observed in the wild-type, WA, and MA1 strains, and no significant Atg8-PE band was probed in the Δ*Bbatg3*, MA2, and MA3 strains. In 2-day-old submerged mycelia from Sabouraud dextrose broth (SDB), Atg8-PE band was only seen in the wild-type strain and not in any other tested strains. Under starvation stress, the lipidation increasing with the stress time was observed in the wild-type, WA, and MA1 strains. However, no lipidation occurred in the Δ*Bbatg3*, MA2, and MA3 strains. These results indicated that the phosphorylation site S136 is essential for Atg-PE conjugation.

## DISCUSSION

The autophagic pathway has been linked to a wide variety of physiological processes in filamentous fungi, including conidiation, vegetative growth, fruiting-body formation, life span, and pathogenicity (5). In B. bassiana, autophagy occurs throughout its life cycle, and the ATG1 gene is involved at various stages of fungal development and virulence (6). The present investigation revealed that the kinase Atg1 has a comprehensive function in the proteome and phosphoproteome during fungal growth and development under aerial conditions.

To our knowledge, this study presents the largest Atg1-dependent phosphoproteome analysis of filamentous fungi. In B. bassiana, Atg1 preferentially phosphorylates serine residues in target proteins similar to that in S. cerevisiae (21). Cdc14 (a dual-specificity phosphatase) contributes to asexual development and stress responses in B. bassiana through dephosphorylation reactions and has a preferential reaction site for serine (27). This implies that B. bassiana develops serine as the preferential site for transduction of phosphorylation signals, although more substrates of various kinases and phosphatases need to be investigated. As for screening the phosphorylation sites, two strategies had been utilized. Papinski et al. (20) and Egan et al. (24) used a strategy of screening the peptide library, in which the data indicated the direct phosphorylation sites. Hu et al. (21) and we used a strategy of analyzing the comparative phosphoproteomics, in which the data indicated the Atg1-dependent phosphoproteome. Thus, there is comparability between our findings and those revealed by Hu et al. (21). The Atg1-dependent phosphoproteome in B. bassiana differs from that in S. cerevisiae (21). In yeast Atg1 targets, serine acts as a phosphorylation acceptor, and hydrophobic amino acids (i.e., leucine and methionine) have higher frequency at the position −3 of phosphorylation site than serine (20). The predicted substrates of BbAtg1 significantly differ from those revealed for yeast, fruit flies, and humans (20, 23, 24). This suggests that the substrate diversity might be due to the evolution of the Atg1 kinase signaling pathways among different eukaryotes. Additionally, Atg3 was verified to be a direct phosphorylation target of Atg1 via *in vitro* kinase assay. The serine acceptor and “hydrophobic” amino acid (L) at position −3 were still observed in BbAtg3, which fits the Atg1 consensus sequence revealed previously (20, 24). This property is conserved in the model insect- and plant-pathogenic fungi. In the saprobe N. crassa, Atg3 had methionine at the position −3. No conservative property is present in Aspergillus species, yeast, or higher eukaryotes. These results indicate that the phosphorylation site in Atg3 from entomopathogenic fungi has a closer relationship with that of plant-pathogenic fungi than that from *Neurospora* and Aspergillus species. This trend is similar to the phylogenetic relationship among these fungi (28, 29), implying that the phosphorylation site in Atg3 potentially evolves with the divergence among filamentous fungi. Additionally, B. bassiana has a more distant relationship with yeast and higher eukaryotes, which explains the fact that the phosphosite of BbAtg3 is not present in Atg3 from these organisms. Atg3 acts as an evolutionarily conserved E2-like enzyme during autophagic process (11). These results suggest that eukaryotes might evolve different regulatory mechanisms that control Atg3 activity. From an evolutionary point of view, it is conserved that Atg1 strongly prefers serine over threonine as the phosphorylation site, and the Atg1 substrates are of great diversity. As insufficient motif information is available for the substrates of Atg1/ULK, it is difficult to examine the diversity of motif distribution among different organisms. However, some of the motifs identified in this study are present in other fungi and pathways. In Aspergillus flavus, MAP kinase kinase kinase Ste11 is required for vegetative growth and conidial production (30), and its five motifs (i.e., SDXE, SP, PXSP, RXXS, and TP) are present in the Atg1-dependent phosphoproteome of B. bassiana. In yeast, Hog1 acts as a kinase in the MAPK cascade, and cAMP-dependent protein kinase (CDK) is also an essential regulator. Two motifs (i.e., SP and TP) were also observed in the Hog1- and CDK-dependent phosphoproteome of S. cerevisiae and Cryptococcus neoformans, respectively (31, 32). In B. bassiana, the MAPK and CDK pathways significantly regulate mycelial growth and asexual sporulation (33, 34). This suggests that in B. bassiana, Atg1 may potentially interact with MAPK and other pathways during fungal differentiation. Furthermore, some motifs appear in distantly related organisms. In Arabidopsis thaliana, for instance, the motif (SP) also corresponds to the targets of the kinases from the CDK and MAPK families (35). Together, these findings not only reinforce the fact that most kinase-substrate motifs are extensively distributed in eukaryotes (36) but also suggest that certain motifs are evolutionarily conserved in the substrates of different kinases.

On the aerial surface, filamentous fungi develop robust mycelia on which numerous conidia mature. The conidiation process aids fungal dispersal, survival, and evolution in the natural environment (37, 38). In addition to its involvement as a “core” machinery in autophagy initiation (12), a well-known physiological role of Atg1 in filamentous fungi is to regulate conidial development. This phenomenon has been observed in many fungi, such as Aspergillus oryzae, B. bassiana, and *M. grisea* (10, 16, 39). In *M. grisea*, glycogen autophagy contributes to conidiation. Exogenous nutrients (e.g., glucose and glucose-6-phosphate) restore conidiation in autophagy-null mutants (17). Given the importance of autophagy in mobilizing and transferring nutrients, the role of Atg1 in conidiation is considered to be the result of autophagy mediated by Atg1. This study revealed that the Atg1-mediated phosphoproteome was mainly associated with signal transduction (e.g., MAPK signaling pathway), metabolism (e.g., starch and sucrose metabolism), the cell cycle, and autophagy. For example, the MAPK signaling pathway involves a series of serine-threonine protein kinases. In *M. grisea*, ATG1 phosphorylates MAP kinase kinase (Mkk1), a key component of the cell wall integrity pathway (40). In B. bassiana, the MAPK pathway plays an important role in asexual conidiation (33). These findings suggest that Atg1 interacts with other signaling transduction pathways. In B. bassiana, many conidiation-related genes are associated with autophagy (e.g., *BbATG5*), the cell cycle (e.g., *BbCdc14*), and metabolism (e.g., *BbSNF1*) (25, 41, 42). Additionally, Atg1 regulates the conidial levels of the conidiation-related protein BbCP15 in B. bassiana (10). Thus, in terms of function, the kinase activity of Atg1 has a global role in various physiological processes in B. bassiana, although the detailed mechanisms need to be further elucidated.

The phosphorylation activity of Atg1 is also essential for autophagy in B. bassiana. Notably, B. bassiana Atg1 is required for phosphorylation of Atg2, Atg3, Atg9, and Atg11. In the Δ*Bbatg1* mutant strain, the protein levels of three proteins (Atg2, Atg3, and Atg11) did not change significantly. Thus, these proteins may be regulated only by the phosphorylation activity of Atg1. In budding yeast, Atg1 phosphorylates Atg2, Atg6, and Atg9 (20). Upon initiation of autophagy, dephosphorylated Atg13 binds to Atg1 and then associates with Atg17, Atg29, and Atg31. The resulting complex phosphorylates Atg2, which is required for autophagosome formation under starvation stress (13). In this study, Atg1 contributed to Atg2 phosphorylation during B. bassiana development. Thus, Atg2 is a conservative target of Atg1 in fungi and functions in various biological processes. In B. bassiana, Atg3 phosphorylation by Atg1 is essential for autophagy under oxidative stress and during aerial development. ULCS is required for Atg8 activation and localization in the preautophagosomal structure (PAS), involving two ligation pathways. Atg3 acts as an E2-like enzyme with protease Atg4 and the E1-like enzyme Atg7. This pathway is critical for the formation of Atg8-phosphatidylethanolamine (PE), which is localized in the PAS (13). Our findings reveal that Atg1 regulates ULCS by phosphorylating Atg3 in *B.* bassiana (Fig. 5), and phosphorylation of Atg3 is critical for Atg8 lipidation (Fig. 10). In yeast, Atg9 phosphorylation is required for the recruitment of Atg8 and Atg18 to the PAS and elongation of the isolation membrane, which is essential for the autophagy pathway (20). Atg9, a membrane protein, interacts with Atg2 and localizes to the PAS. In this manner, Atg9 directs or facilitates the delivery of proteins and lipids to the PAS for autophagosome expansion (43, 44). In mammals, BECN1 (ortholog of yeast Atg6) phosphorylation by ULK1 (ortholog of yeast Atg1) enhances the activity of the Atg14-containing phosphoinositide 3-kinase complex (PI3K) that is required for full autophagic induction (45). Meanwhile, ULK1 regulates ATG14 activity via direct phosphorylation (46). These findings focus on the role of Atg1 phosphorylation activity in autophagosome formation and maturation. In budding yeast, Atg29 acts as a common downstream target of rapamycin complex 1 (TORC1) and Atg1 and coordinates their activities during autophagy under nutritional starvation (21). As for B. bassiana Atg11, Atg1 is required for phosphorylation of four amino acid residues and negatively regulates phosphorylation of one amino acid. Atg11 is highly conserved in eukaryotes and acts as an essential scaffold protein that mediates selective autophagy (47). Therefore, in addition to its role as a key component of the autophagic pathway, Atg1 has great potential in the regulation of various components of the autophagy pathway. This suggests that the cross talk between the Atg1 complex and other autophagy machineries is required for coordination of the autophagy process. Future studies are required to comprehensively understand this regulatory mechanism in different organisms and under different stimuli.

In summary, we revealed the global phosphorylation effects of Atg1 in *B.* bassiana via quantitative phosphoproteomic analyses. Through integration of phosphoproteomic and proteomic data, we generated a database of potential targets that are significantly regulated by Atg1 phosphorylation. Further, we demonstrated that Atg1 regulates Atg3 phosphorylation, which is essential for autophagy. This finding suggests that Atg1 not only initiates autophagy but also orchestrates the autophagic pathway by its kinase activity. Our study may serve to guide future research on the evolutionary diversification of the Atg1 kinase signaling pathways among different pathogenic fungi.

## MATERIALS AND METHODS

### Microbial strains and culture conditions.

The wild-type (WT) strain of *B.* bassiana, ARSEF 2860, and its mutant strains were maintained as described previously (47). Sabouraud dextrose agar (SDA; 4% glucose, 1% peptone, and 1.5% agar; with 1% yeast extract [SDAY]) was used as an enriched medium to culture the fungal strains. Czapek-Dox plates (CZA; 3% glucose, 0.3% NaNO_3_, 0.1% K_2_HPO_4_, 0.05% KCl, 0.05% MgSO_4_, and 0.001% FeSO_4_ plus 1.5% agar) was used as the medium for screening transformants and for phenotypic analyses.

### Sample preparation for proteomic and phosphoproteomic analyses.

The Δ*Bbatg1* mutant strain was constructed as described in a previous study (10). Comparative proteomic and phosphoproteomic analyses were performed between the WT and disruption mutant (DM) strains as previously described (27). Fungal strains were cultured on SDAY plates for 3 days at 25°C. Each strain had two independent biological replicates. The resultant mycelia (200 mg) were ground into a powder in liquid nitrogen and mixed with 1.2 mL lysis buffer, consisting of 8 M urea, 50 mM Tris-HCl (pH 8.0), 1% NP-40, 1% sodium deoxycholate, 2 mM EDTA, 5 mM dithiothreitol (DTT), 1% protease inhibitor (Sigma; P8215), and 1% phosphatase inhibitor (Roche, Mannheim, Germany). The mixture was sonicated on ice, and the protein solution was obtained by centrifugation at 20,000 × *g* at 4°C for 10 min. Protein concentration was determined using a 2-D Quant kit, and the protein integrity was assayed by sodium dodecyl sulfate-polyacrylamide gel electrophoresis (SDS-PAGE).

For tryptic digestion, 300-μg and 2-mg quantities of proteins were used for proteomic and phosphoproteomic analyses, respectively. The extracted proteins were reduced with 5 mM DTT for 45 min at 45°C and alkylated with 30 mM iodoacetamide (IAA) for 1 h at 25°C in the dark. The resulting protein was collected and dissolved in 0.1 M triethylammonium bicarbonate (TEAB) solution. Proteins were digested overnight with trypsin (1:50 trypsin-protein) at 37°C.

After enzymatic hydrolysis, the resulting peptides were desalted on a C_18_ SPE column (Phenomenex), vacuum dried, and redissolved in 0.5 M TEAB. Aliquots (100 μg) of the peptides were labeled with a TMT kit (Thermo Fisher Scientific, Waltham, MA) at ambient temperature for 2 h. The labeled peptides were mixed and fractionated on a C_18_ column. Finally, for the proteomic assay, the peptides were grouped into 20 fractions; for the phosphoproteomic assay, the peptides were grouped into 8 fractions. All fractions were dried by vacuum centrifugation.

For phosphopeptide enrichment, the trypsin-hydrolyzed proteins were dissolved and incubated with titanium oxide (TiO_2_) beads (28-9440-10; GE Healthcare) for 1 h at 25°C. After centrifugation, the peptides were eluted from the beads, and the eluates were desalted and vacuum dried.

### Liquid chromatography-tandem mass spectrometric (LC-MS/MS) analysis.

Phosphoproteome and proteome samples were analyzed at Micrometer Biotech (Hangzhou, Zhejiang, China). Peptides were dissolved in 0.1% formic acid (FA) (solution A) and then separated at a constant flow rate (250 μL/min) of solution B (0.1% FA in 98% acetonitrile [ACN]) on a reversed-phase analytical column (Acclaim PepMap RSLC; Thermo Fisher Scientific), installed on an Ultimate RSLCnano 3000 system. The solution B gradient was programmed as follows: increasing from 2% to 10% within the first 6 min, from 10% to 20% from 7 to 50 min, reaching 80% from 51 to 57, holding at 80% from 58 to 61 min, decreasing to 2% within 1 min, and holding at 2% for the last 7 min. The resolved peptides were analyzed using a Q Exactive HF hybrid quadrupole-Orbitrap mass spectrometer (Thermo Scientific) at an electrospray voltage of 2.0 kV. The range for the full-scan mass spectrum was set from 350 to 1,800 *m/z*. The intact peptides were first detected at a resolution of 60,000 *m/z*, and the ion fragments were analyzed at a resolution of 15,000 *m/z*. The mass spectrometric proteomics data have been deposited at the ProteomeXchange Consortium (http://proteomecentral.proteomexchange.org) via the iProX partner repository (48) with the data set identifier PXD023499.

### Protein/peptide identification and quantification.

The raw data were converted into Mascot generic files with Proteome Discoverer (version 1.4.1.14; Thermo Scientific). Proteomes and phosphoproteomes were searched against the B. bassiana proteome database (UniProt Proteome identifier [ID] UP000002762) using Mascot (version 2.3.02; Matrix Science) and MaxQuant (version 1.5.2.8; Matrix Science), respectively. The parameters for the data search were set according to the manufacturer’s instructions.

To identify DAPs and DAPPs, the relative abundances of proteins/phosphopeptides between the WT and DM strains were analyzed as described previously (49, 50). In brief, only unique proteins/phosphopeptides were used for the quantitative comparison. The reliability of two biological repeats was evaluated by calculating the multivariate Pearson correlation coefficients (51). Mascot software was used to calculate the intensity ratios of the TMT reporter ion (IRRI), which indicated the relative abundance of paired proteins/phosphopeptides. Statistical analysis was performed using Student’s *t* test on the IRRI values between the WT and DM strains using the threshold of *P *value of <0.05. DAPs and DAPPs were considered when the fold change was >1.50 or <0.67. The fold changes were subdivided into four ranks: Q1 (0 to 0.5), Q2 (0.5 to 0.67), Q3 (1.5 to 2.0), and Q4 (>2.0).

To determine the effect of phosphorylation alone, we used the following criteria: (i) phosphopeptides were reduced >1.5-fold in the Δ*Bbatg1* mutant strain and (ii) their protein levels did not change significantly or changed no more than >1.5-fold.

### Bioinformatic analyses of the identified proteins and phosphoproteins.

To functionally classify DAPs and DAPPs, the proteins and phosphopeptides were first annotated with the category of Gene Ontology (GO). Enrichment analyses were performed using a two-tailed Fisher exact test, and correction for multiple-hypothesis testing was carried out using standard false-discovery rate control methods. The enriched categories were determined when the corrected *P* value was <0.05.

For phosphorylation motif analysis, Motif-X software was used to discover the sequence model near the specific positions in all identified phosphorylated proteins. Sequences were centered at their phosphorylation site and extended to 13 amino acids (±6 residues). All protein sequences in the database were used as the background. All parameters in the software were set as defaults, and the threshold value for statistical significance was set to 10^−6^. To determine the overpresentation of the motif, all differentially altered phosphorylation sites (DASs) were appointed a motif. A two-tailed Fisher exact test was also used to explore motif enrichment of each rank of DAPP against all identified motifs, using a corrected *P* value of <0.05 as the threshold.

According to the phosphorylation site information of the Atg1 substrates in yeast (20), the potential Atg1 substrates in B. bassiana were retrieved in the repressed DAPPs (ranks Q1 and Q2). The resultant B. bassiana proteins were used as queries to search their homologs in yeast, fruit flies, and humans.

### Disruption and complementation of Atg3 in *B.* bassiana.

Phosphorylation of Atg3 is affected byAtg1 protein. The physiological roles of Atg3 in B. bassiana were studied using gene disruption and complementation, as described previously (47). All primers used in this study are listed in Table S1. The 5′ and 3′ regions of the *BbATG3* open reading frame (ORF) were amplified by PCR using the corresponding primer pairs P1/P2 and P3/P4, respectively. The resulting fragments were separately cloned into the EcoRI/BamHI and XbaI/HpaI sites of p0380-bar with ammonium glufosinate resistance gene, using the ClonExpress II one-step cloning kit (Vazyme Biotech, Nanjing, China). The resultant plasmid was designated p0380-BbAtg3-KO for target gene replacement. For gene complementation, the complete *BbATG3* ORF together with its partial up- and downstream sequences was amplified with the primer pair P7/P8. The PCR fragments were cloned into the vector p0380-sur-gateway (conferring resistance to chlorsulfuron). The complementation plasmid was named p0380-sur-BbAtg3. After fungal transformation, the transformants were primarily verified by PCR with the primer pair P5/P6. Further validation was performed via Southern blot analysis using a digoxin (DIG) DNA labeling and detection kit (Roche, Germany). The templates (260 bp) were amplified with the primer pair P9/P10 and used in probe preparation with the digoxin-labeling method.

10.1128/msystems.01463-21.10 TABLE S1 Primers used in this study. Download Table S1, DOCX file, 0.02 MB. Copyright © 2022 Lin et al. 2022 Lin et al. https://creativecommons.org/licenses/by/4.0/ This content is distributed under the terms of the Creative Commons Attribution 4.0 International license.

### Visualization of the autophagic process.

The autophagic process was visualized using transmission electron microscopy (TEM) and laser scanning confocal microscopy (LSCM) (47). For TEM, conidia were grown in Sabouraud dextrose broth (SDB) at 25°C. Two-day-old mycelia were washed with water and then subjected to starvation in Czapek-Dox medium minus carbon and nitrogen source for 3 h. The stressed cells were initially fixed in glutaraldehyde, dehydrated in ethanol, and infiltrated with resin. The ultrathin sections were poststained in uranyl acetate and lead citrate and viewed with an H-7650 transmission electron microscope (Hitachi).

For LSCM, the autophagic process was visualized using the fusion protein BbAtg8-GFP (10). The pyrithiamine resistance gene (*ptrA*) was amplified from pME2892 (52) with primers P11 and P12, and used to replace the bar cassette in plasmid p0380-TB, generating plasmid p0380-ptrA. The expression cassette of *BbATG8-GFP* was amplified with primers P13 and P14, and the resultant fragment was cloned into plasmid p0380-ptrA. The resulting plasmid p0380-GA8-ptrA was introduced into the WT and Δ*Bbatg3* mutant strains. The transformants were screened on CZA plates supplemented with pyrithiamine as the selection agent, generating WT-GA8 and A3-GA8 strains. To visualize autophagy under starvation, 2-day-old mycelia from SDB media were stressed as mentioned above for 3 h. To visualize autophagy during development, conidia were inoculated onto SDAY plates at 25°C for 3 days. Samples were stained with fluorescent dye FM4-64 specific to the membrane, and dual-color fluorescence was detected using an LSCM (LSM 710; Carl Zeiss Microscopy GmbH, Jena, Germany).

### Phenotypic evaluation.

Phenotypic assays were performed as described previously (47). All assays were performed with the indicated strains in triplicates. To generate chemical stress in the fungi, the following chemicals were included in the CZA plates supplemented with menadione (0.02 mM; working concentration). Aliquots of 1-μL suspensions (1 × 10^6^ conidia/mL) were dotted onto the plates and cultured at 25°C. Colony diameters were measured 7 days postincubation (dpi). For aerial sporulation, a conidial suspension (100 μL of 10^7^ cells/mL) was inoculated onto SDAY plates at 25°C. The mycelial discs were sampled at 8 dpi, and their conidia were quantified. Conidial yield is presented as the spore number per square centimeter.

### Phosphorylation assays for BbAtg3.

Phosphorylation sites were predicted at Ser^135^ and Ser^136^ located in the Autophagy_N domain. To determine the requirement of BbAtg1 for phosphorylation, *BbATG3* was amplified by PCR with the primer pair P15/P16. The DNA fragment was fused to the N terminus of mCherry in plasmid pTEF-mcherry-sur, in which the hybrid gene was under the control of a translation elongation factor (TEF) promoter. The resultant plasmid was transformed into the WT and Δ*Bbatg1* mutant strains, and the transformants were screened on CZA plates containing chlorsulfuron.

To validate the roles of the phosphorylation sites in BbAtg3, the Ser was converted into Ala via point mutation using overlapping-extension PCR (53). The primers used for gene cloning (P15/P16) and mutation (P17 to P22) are shown in Table S1. The two serine sites (S135 and S136) were mutated individually and simultaneously. The WT and mutated *BbATG3* genes were amplified and cloned into plasmid pTEF-mcherry-sur. The resulting plasmid was transformed into the Δ*Bbatg3* mutant strain, and the transformants were screened as described above. The strains obtained for the WT and mutated *BbATG3* genes were designated WA, MA1, MA2, and MA3, respectively. The Δ*Bbatg3* mutant strain (DS) and the Δ*Bbatg3* mutant with the wild-type *ATG3* (WA) were used as the controls in phenotypic assays.

Western blot analysis was used to detect the phosphorylated proteins. Fungal strains were cultured on SDAY plates at 25°C for 3 days. Cell lysates were prepared by grinding the mycelia in liquid nitrogen with phosphate-buffered saline (PBS). After clearing the cell debris and quantifying the proteins, 100 μg of proteins was separated by SDS-PAGE and transferred to a polyvinylidene fluoride (PVDF) membrane. After blocking with 1% bovine serum albumin, the membrane was probed with anti-mCherry primary antibody (SAB2702286; Sigma) and horseradish peroxidase (HRP)-conjugated goat anti-mouse secondary antibody (BA1050; Boster Biological Technology, Wuhan, China). The phosphorylated protein was probed with biotinylated Phos-Tag (Wako Pure Chemical Industries, Osaka, Japan), and then biotin was detected with HRP-conjugated streptavidin (RPN1231; GE Healthcare Bio-Sciences). Protein bands were visualized using the chemiluminescence method.

### Assay for *in vitro* kinase activity of BbAtg1.

A strategy of endogenous preparation of functional Atg1 (20) was adopted in this study. BbAtg1 was prepared endogenously in the Δ*Bbatg1* mutant strain, using GFP-Atg8 as the reporter to indicate the functional state of BbAtg1. *BbATG1* was amplified by PCR with the primer pair P23/P24, generating a fusion gene with the human influenza virus hemagglutinin (HA) and Flag gene at its 5′ and 3′ termini, respectively. The hybrid gene was transformed into the Δ*Bbatg1* mutant strain. The resultant transformant was grown in SDB for 2 days, and the mycelia were exposed to starvation for 3 h. The harvested mycelia were rinsed and ground in liquid nitrogen. The disrupted cells were thawed in lysis buffer (50 mM Tris HCl, 150 mM NaCl [pH 7.5]) supplemented with protease inhibitor cocktail (Roche), 20 μM MG132 (Selleckchem, TX), 1 mM phenylmethylsulfonyl fluoride (Amresco, OH) and phosphatase inhibitor mixture (KeyGen Biotech, Nanjing, China). Mycelial debris was removed by centrifugation. BbAtg1 was purified through immunoprecipitation with anti-FLAG M2 magnetic beads (M8823; Sigma-Aldrich) and anti-HA agarose (26181; Thermo Scientific, IL). BbAtg3 and its mutated forms were prepared in a bacterial expression system as described previously (54). Their coding sequences were amplified using the primer pair P25/P26 (Table S1) and cloned into the BamHI and XhoI sites in the pET28a expression vector (Novagen) (54). Expression plasmid was transformed into the E. coli Rosetta DE3 strain. The protein was purified through Ni-chelating affinity. All manipulations for purification were performed according to the manufacturer’s instructions.

Assays for *in vitro* phosphorylation reaction were performed as described previously (55). Briefly, BbAtg3 was incubated with BbAtg1 in 50 μL of reaction buffer (0.5 mM DTT, 10 mM MgCl_2_, 2 mM MnCl_2_, and 50 mM ATP in 10 mM Tris-HCl [pH 7.8]) for 3 h at 25°C. The reaction products were resolved on an SDS-PAGE gel, and the phosphorylated proteins were probed with biotinylated Phos-Tag (Wako Pure Chemical Industries, Osaka, Japan) as mentioned above.

Additional homologs of Atg3 were downloaded from the NCBI database (http://www.ncbi.nlm.nih.gov/), and their phylogenetic relationships were analyzed with MEGA version 5 software (56).

### Protein interaction between BbAtg1 and BbAtg3.

Yeast two-hybrid (Y2H) assays were used to determine the direct interaction between two proteins using a Matchmaker GAL4 Two-Hybrid System 3 kit (Clontech Laboratories, CA). In brief, *BbATG1* and *BbATG3* were amplified with the primer pairs P27/P28 and P29/P30, respectively. These two fragments were cloned into the vectors pGADT7 and pGBKT7, respectively. The resulting plasmids were transformed into yeast YH109. Coexistence of two plasmids was validated by culturing the yeast transformants on an SD/Leu-Trp plate. The positive interaction was determined when the yeast transformants grew well on SD/Trp-Leu-His-Ade medium.

Bimolecular fluorescence complementation (BiFC) assay was used to probe *in vivo* interaction between two proteins. The YFP gene was split into the N (YN) and C termini (YC) as the reporter for protein interaction. The promoter region of translation elongation factor 1 (TEF1) of *B.* bassiana and *trpC* terminator of Aspergillus nidulans were amplified with primer pairs P31/P32 and P33/P34, respectively. The resultant fragments were ligated into plasmid p0380-TB, generating the plasmid p0380-TEF-MCS-TER-bar (pTMTB). The YC fragment was amplified using vector pUC35S-nYFP/C (Biogle Co., Hangzhou, China) as a template. Multiple-cloning sites (MCS) were introduced downstream and upstream of YC with primer pair P35/P36. The obtained fragments were cloned into pTMTB, generating the plasmid p0380-TEF-MCS-YC-bar (p0380TM-YC-B). YN was amplified from pUC35S-cYFP/N (Biogle Co.) with primer pair P37/P38 and integrated into plasmid p0380-TEF-sur (57), generating p0380-TEF-MCS-YN-sur (p0380TM-YN-S). To test the *in vivo* protein interaction, the primer pairs P39/P40 and P41/P42 were used to amplified *BbATG1* and *BbATG3*, respectively. The amplified fragments were cloned into p0380TM-YC-B and p0380TM-YN-S, respectively. The resultant plasmids were named as p0380T-ATG1-YC-B and p0380T-ATG3-YN-S, and these were successively transformed into the wild-type strain of B. bassiana. The transformants were propagated in SDB medium and starved as described above. The expression of *YC*-*BbATG1* and *YN*-*BbATG3* was established with immunoblotting assay, using anti-HA and anti-myc antibody, respectively. Actin was used as an endogenous reference. Fluorescent signals in mycelia were observed under a fluorescence microscope (LSM 710; Carl Zeiss Microscopy GmbH, Jena, Germany). Plasmids containing YC or YN were used as the negative controls. All primers are listed in Table S1.

### Phenotypic validation of phosphorylation sites in BbAtg3.

Phenotypic assays, including fungal resistance, oxidative stress, and conidiation, were performed with the WA, MA1, MA2, and MA3 strains, using a strain expressing only *mCherry* as a control. To visualize autophagic flux, plasmid p0380-GA8-ptrA was transformed into the WA, MA1, MA2, and MA3 strains, and the transformants were screened on the CZA plates containing pyrithiamine as the selection agent. The resultant strains were cultured on SDAY plates for 5 days, and the fluorescent signals were examined under a laser scanning confocal microscope.

### Assay for Atg8 lipidation.

Atg8 lipidation assay were performed as described previously (58). In brief, the plasmid p0380-GA8-ptrA was transformed into the WA, MA1, MA2, and MA3 strains as mentioned above. The plasmid was transformed into the wild-type and Δ*Bbatg3* mutant strains, and the resultant strains were used as positive and negative controls, respectively. The wild-type strain was included as a blank control. Aerial mycelia were cultured on SDAY plate for 4 days, and submerged mycelia were grown in SDB medium for 2 days. For starvation, the submerged mycelia were rinsed and stressed in mineral solution for 1, 3, and 6 h. The conjugation of Atg8 to phosphatidylethanolamine (PE) was analyzed on by SDS-PAGE with urea, and the proteins were detected by Western blotting with mouse monoclonal enhanced GFP (eGFP) antibody (TA150052; OriGene, MD, USA). Actin was used as an endogenous reference and probed with rabbit anti-actin antibody (FD0060, Fdbio science, Hangzhou, China). HRP-conjugated goat anti-mouse and anti-rabbit IgG was used as the secondary antibody (BA1050; Boster Biological Technology, Wuhan, China).

### Statistical analysis.

Tukey’s honestly significant difference (Tukey’s HSD) test was used to determine the significance of the indicated phenotypes among different strains.

### Data availability.

The mass spectrometric proteomics data have been deposited at the ProteomeXchange Consortium (http://proteomecentral.proteomexchange.org) via the iProX partner repository with the data set identifier PXD023499.
