# Supplementary figures and images for: Proteomic and Phosphoryproteomic Investigations Reveal that Autophagy-Related Protein 1, a Protein Kinase for Autophagy Initiation, Synchronously Deploys Phosphoregulation on the Ubiquitin-Like Conjugation System in the Mycopathogen Beauveria bassiana
Source: mSystems. 2022 Feb 8;7(1):e01463-21. doi: 10.1128/msystems.01463-21 (PMC8823290; doi:10.1128/msystems.01463-21)

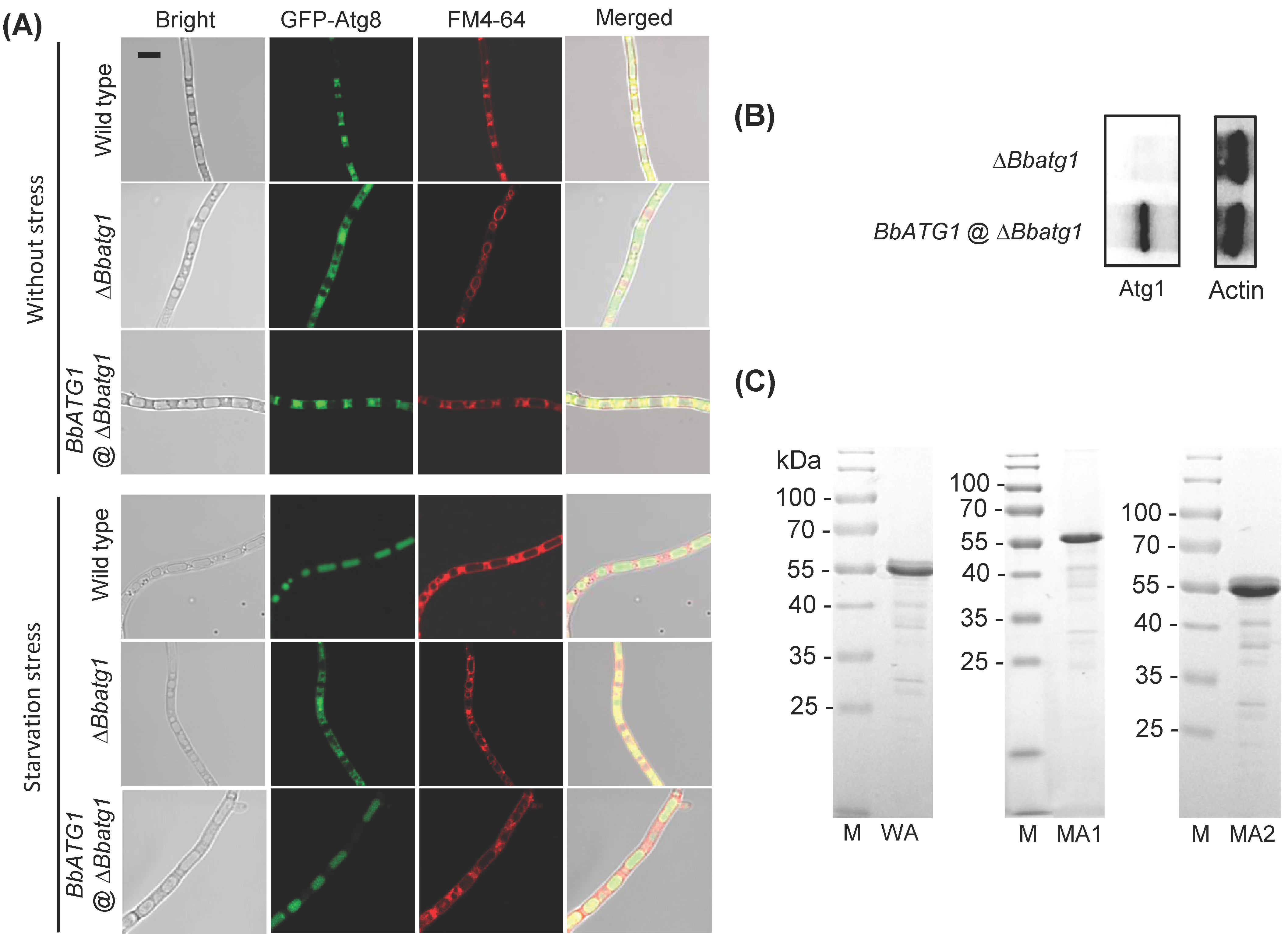

Supplement: FIG S1 [file msystems.01463-21-sf001.tif]

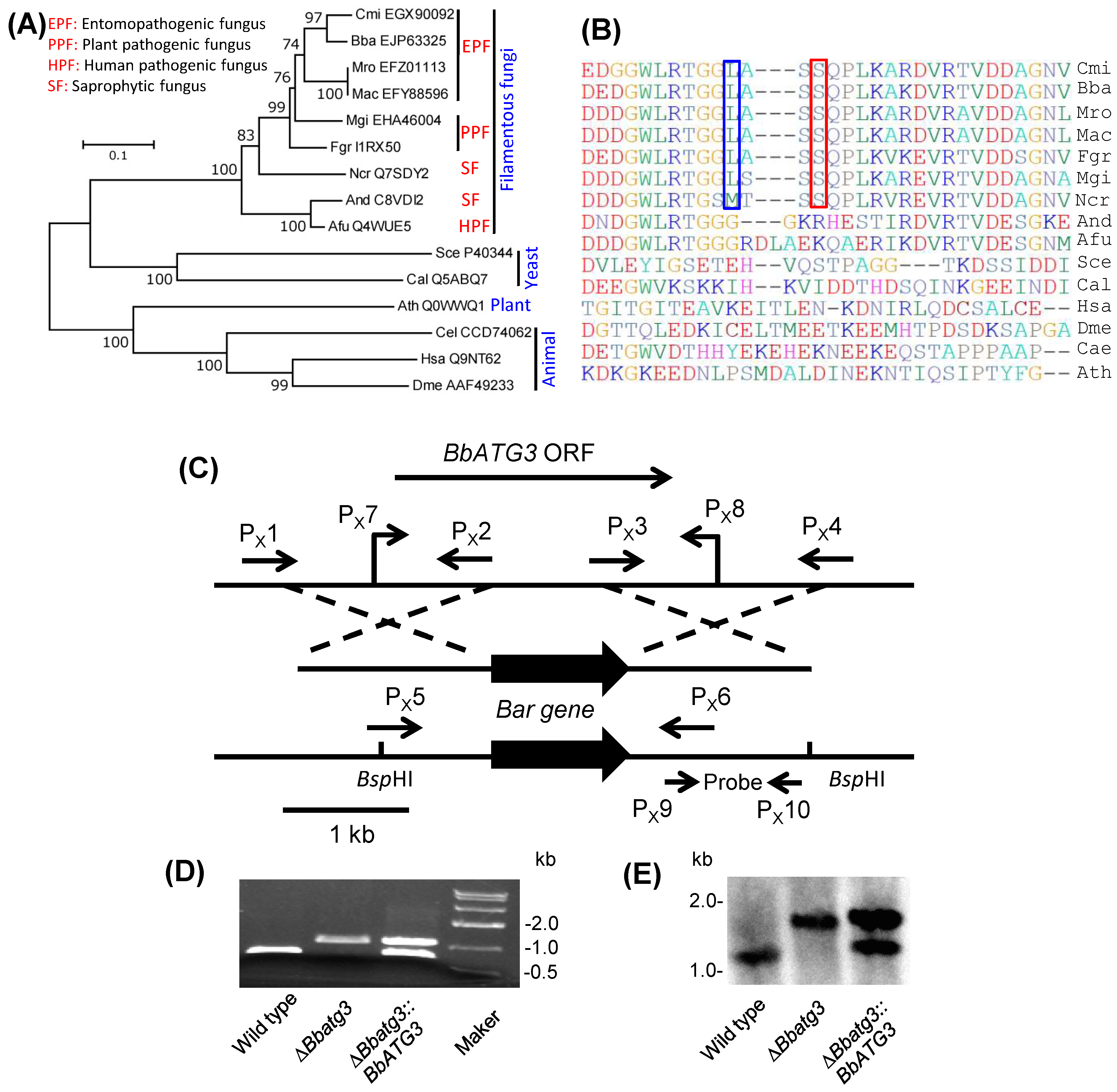

Supplement: FIG S2 [file msystems.01463-21-sf002.tif]
