# Supplementary material for: Proteomic and Phosphoryproteomic Investigations Reveal that Autophagy-Related Protein 1, a Protein Kinase for Autophagy Initiation, Synchronously Deploys Phosphoregulation on the Ubiquitin-Like Conjugation System in the Mycopathogen Beauveria bassiana
Source: mSystems. 2022 Feb 8;7(1):e01463-21. doi: 10.1128/msystems.01463-21 (PMC8823290; doi:10.1128/msystems.01463-21)
Supplement: TABLE S1 [file msystems.01463-21-st001.docx]

**Supplementary information**

**Proteomic and phosphoryproteomic investigations reveal that autophagy-related protein 1, a protein kinase for autophagy initiation, synchronously deploys phospho-regulation on ubiquitin-like conjugation system in mycopathogen *Beauveria bassiana***

**Hai-Yan Lin, Jin-Li Ding, Yue-Jin Peng, Ming-Guang Feng and Sheng-Hua Ying***

*Institute of Microbiology, College of Life Sciences, Zhejiang University, Hangzhou, 310058, China*

***Corresponding author:** Sheng-Hua Ying (e-mail: yingsh@zju.edu.cn), Institute of Microbiology, College of Life Sciences, Zhejiang University, Hangzhou, China, 310058; Tel: 86-571-88206199, FAX: 86-571-88206178.

**Table S1 Primers used in this study.**

| **Primer name** | **Primer sequences (5´-3´)*** | **Purpose** |
| --- | --- | --- |
| P1 | TGGGCCCGGCGCGCCGAATTCGTGGCGAGCATTTACTACTG | Amplifying upstream  flanking sequence |
| P2 | TGGCTGCAGGTCGACGGATCCGATGCCTCTATGATTATGTTCT |  |
| P3 | GACCCATGGCTCGAGTCTAGATTCCTTACCAGCAAACACC | Amplifying downstream  flanking sequence |
| P4 | GGTGGTGGTGGCTAGCGTTAACACCCTTGAGCCATCCATA |  |
| P5 | CCACCGATAGATACAAATACCA | Confirming the candidate transformants |
| P6 | TGTAGAGGGTGTATGTGCGTC |  |
| P7 | ATCCGTCGACCTGCAGCCAAGCTTTGGGATACCAACACTAAGAA | Obtaining the entire gene for complementation |
| P8 | ACACTAGTCAGATCTTCTAGTGTGTGAGCACAACCCGACTA |  |
| P9 | TTCCTTACCAGCAAACACC | Preparing the probe template |
| P10 | GTGACGGTCTTGTCCTTGT |  |
| P11 | CGTCGACCTGCAGCCAAGCTTGGGCAATTGATTACGGGATC | Amplifying pyrithiamine resistance gene |
| P12 | CACTAGTCAGATCTTCTAGAATGGGGTGACGATGAGCCGC |  |
| P13 | GGGCCCGGCGCGCCGAATTCCGTTCTTCCGAATAGCATC | Amplifying *BbATG8-GFP* cassette |
| P14 | AATCAATTGCCCAAGCTTGGTCGACCAAGTCCCTCCAC |  |
| P15 | CAATCACAAACACCTTCAAAATGAACTATATCTACTCAACAGTCA | Amplifying upstream of *BbATG3* |
| P16 | CTCGCCCTTGCTCACCATCACTCCCATAGTGAAATCGTGCTCG | Amplifying downstream of *BbATG3* |
| P17 | CTTGAGCGGCTGCGAGCCCGCCAGGCCGCC | Mutation of S135 |
| P18 | GGCGGCCTGGCGGGCTCGCAGCCGCTCAAG |  |
| P19 | CTTGAGCGGCTGCGCGCTCGCCAGGCCGCC | Mutation of S136 |
| P20 | GGCGGCCTGGCGAGCGCGCAGCCGCTCAAG |  |
| P21 | CTTGAGCGGCTGCGCGCCCGCCAGGCCGCC | Double mutation of S135 and S136 |
| P22 | GGCGGCCTGGCGGGCGCGCAGCCGCTCAAG |  |
| P23 | ATCACAAACACCTTCAAAATGTACCCTTACGATGTTCCTGATTACGCT ACTAGCCGACAGGAAGGGGCCT | Expression of *BbATG1* |
| P24 | GTCGTGATCCTTGTAGTCCGTTGAGCCATAGGACGGCACAC |  |
| P25 | AAAAAGGATCCATGAACTATATCTACTCAACAGTCA | Expression of *BbATG3* and its mutants |
| P26 | AAAAACTCGAGCACTCCCATAGTGAAATCGTGCTCG |  |
| P27 | GCCATGGAGGCCAGTGAATTCATGACTAGCCGACAGGAAGGGGCCT | Amplifying *BbATG1* for Y2H test |
| P28 | CAGCTCGAGCTCGATGGATCCTCACGTTGAGCCATAGGACGGCACA |  |
| P29 | ATGGCCATGGAGGCCGAATTCATGAACTATATCTACTCAACAGTCA | Amplifying *BbATG3* for Y2H test |
| P30 | CGCTGCAGGTCGACGGATCCTTACACTCCCATAGTGAAATCGTGC |  |
| P31 | ACCATGTTGGGCCCGGCGCGCCTTGCACCCAATACGATTAGATTCTT | Amplifying *TEF1* promoter |
| P32 | TGCAGGTCGACGGATCCCCGGGTTTGAAGGTGTTTGTGATTGA |  |
| P33 | AGCCAAGCTTGGTACCGAGCTGATCCACTTAACGTTACTGAAATC | Amplifying terminator |
| P34 | CCGTCACCGAGATTTGACCATGGGAGTGGAGATGTGGAGTGGGC |  |
| P35 | GCATTCAATCACAAACACCTTCAAACCCGGGGATCCGTCGACCTGCAG | Amplifying C-terminal fragment of YFP |
| P36 | ATTTCAGTAACGTTAAGTGGATCTTACTTGTACAGCTCGTCCATGC |  |
| P37 | CTTCAAACCCGGGGATCCGTCGACATGGAGCAAAAGTTGATTTCTGAG | Amplifying N-terminal fragment of YFP |
| P38 | AACGTTAAGTGGATCGGCTGCAGTTAGGCCATGATATAGACGTTGTG |  |
| P39 | CAATCACAAACACCTTCAAAATGACTAGCCGACAGGAAGGGGCCT | Amplifying *BbATG1* for BiFC |
| P40 | GGAACATCGTATGGGTACATCGTTGAGCCATAGGACGGCACACTC |  |
| P41 | TCGCATTCAATCACAAACACCTTCAAAATGAACTATATCTACTCAACAGTCA | Amplifying *BbATG3* for BiFC |
| P42 | CAACTTTTGCTCCATGTCGACGGATCCCACTCCCATAGTGAAATCGTGCTCG |  |

*: The underlined region in primer is identical to that in the target plasmid and required for the recombination reaction in gene cloning. Sequences with double underline are the recognition sites of restriction enzymes.
